# Mimicking Outdoor Ion Migration in Perovskite Solar Cells: A Forward Bias, No-Light Accelerated Aging Approach

**DOI:** 10.1021/acsenergylett.5c00376

**Published:** 2025-03-05

**Authors:** Ulas Erdil, Mark Khenkin, Marko Remec, Quiterie Emery, Vediappan Sudhakar, Rutger Schlatmann, Antonio Abate, Eugene A. Katz, Carolin Ulbrich

**Affiliations:** † Helmholtz-Zentrum Berlin für Materialien und Energie, Hahn-Meitner-Platz 1, 14109 Berlin, Germany; ‡ Faculty of Chemistry, Bielefeld University, 33615 Bielefeld, Germany; § Faculty of Electrical Engineering, University of Ljubljana, 1000 Ljubljana, Slovenia; ∥ Ben-Gurion National Solar Energy Center, Swiss Institute for Dryland Environmental and Energy Research, Jacob Blaustein Institutes for Desert Research, Ben-Gurion University of the Negev, Midreshet Ben-Gurion 84990, Israel; ⊥ Faculty 1 − Energy and Information, Hochschule für Technik und Wirtschaft Berlin, 10313 Berlin, Germany

## Abstract

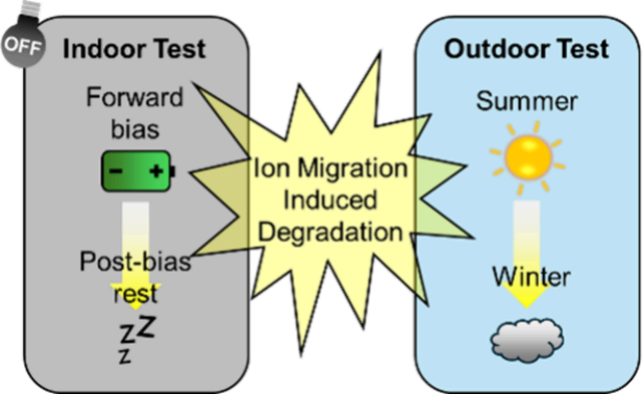

Perovskite solar cells (PSCs) are expected to transform the photovoltaic market; however, their unproven operational stability requires urgent attention, particularly accelerated aging tests. Currently, illumination is the primary stressor in such tests. In this work, we present an accelerated aging procedure consisting of prolonged forward biasing followed by a dark storage (postbias rest) phase, conducted entirely in the dark. During aging under forward bias, ion migration led to impeded charge transport, macroscopic defect growth, and an adverse response of the cells to short light soaking, all of which recovered in the postbias rest phase, yet resulted in increased recombination due to redistribution of ions. We found that outdoor operation of PSCs in Berlin, Germany, over a 20-month period exhibited similar dynamics, with periods of higher temperature and irradiance (spring-summer) aligning with the forward bias phase and cooler, dimmer periods (fall–winter) aligning with the postbias rest phase. This paves the way for accelerated aging tests that can mimic ion migration-induced degradation outdoors without requiring an illumination source.

Although perovskite-based solar cells (PSCs) achieved high efficiency^1^ and promise a low levelized cost of electricity,^2^ the long-term stability of PSCs is still behind that of commercial silicon-based photovoltaic technologies.^3^ Conducting accelerated aging tests, where PSCs are exposed to higher stress levels than encountered at real operation conditions, is a common practice to quickly investigate stability with the ultimate goal of lifetime prediction.^4^ This is inevitable because investigating degradation under real operation conditions requires a significant amount of time as the technology matures, reaching an estimated lifetime of tens of thousands of hours.^5^ However, outdoor tests remain mandatory to validate that the degradation mechanisms and trends simulated via accelerated aging tests are relevant to real operation conditions.^6^ In this direction, the International Summit on Organic Photovoltaic Stability (ISOS) protocols define the frameworks of both indoor and outdoor stability tests of PSCs.^7^

PSCs are susceptible to various stressors and can experience multiple degradation mechanisms,^8,9^ sometimes dominated by a single mechanism^10^ or involving several competing ones.^11^ Perhaps the most common origin for a variety of degradation mechanisms in PSCs is the migration of mobile ions, an intrinsic phenomenon in perovskite absorber.^12^ The soft perovskite structure, combined with its low defect formation energy, readily facilitates ion migration when subjected to stressors^13^ such as illumination,^14−16^ temperature,^17^ and external bias.^18,19^ Given that PSCs will operate at maximum power point (MPP), which corresponds to a forward bias, during outdoor operation,^7^ mobile ions will experience an electric field that drives their migration.^20^ One straightforward way to simulate this bias condition and investigate ion migration-induced degradation is to apply continuous forward bias (positive voltage) in the dark, also referred to as the ISOS-V-1 protocol.^7^

In this direction, Bae and colleagues found that PSCs undergo rapid degradation due to ion migration when the applied voltage is higher than the built-in voltage or open-circuit voltage (*V*_OC_).^21^ Later, Di Girolamo et al. found that continuous forward bias application at 1.2 V leads to accumulation of ions at the interfaces, resulting in partial loss of perovskite crystallinity, also referred to as perovskite amorphization.^22^ Kim et al. even captured this bias-induced amorphization in real time and demonstrated recrystallization when the cells thermally treated.^23^ In addition to migrating across the perovskite absorber, ions have been demonstrated to migrate laterally under forward bias over longer time scales.^24^ Moreover, it is equally important to consider the behavior of PSCs during the period following the removal of stressors, during which both reversible^25,26^ and irreversible processes,^27^ as well as additional loss mechanisms,^28^ have been revealed. These insights are particularly relevant for outdoor operation, where day/night cycles and seasonal variations play a critical role.^29^ While the importance of the dark phases following illumination has been demonstrated,^30−32^ a similar approach has not been explored for voltage, particularly in combination with outdoor tests as a validity check.

In this work, we investigated the effect of continuous forward bias in the dark and subsequent dark storage (postbias rest phase) on the stability of PSCs, probing the underlying mechanisms and, most importantly, implications of the testing sequence for outdoor tests over a period of 20 months. We found that during aging under forward biasing, ions accumulated and immobilized at interfaces and localized sites, leading to impaired charge transport, macroscopic defect growth and an unfavorable response of the cells to brief light soaking. While these adverse effects were reversed during the subsequent postbias rest phase, significant *V*_OC_ losses emerged, attributed to increased recombination. Above all, our findings also revealed that this sequence of mechanisms plays a significant role in outdoor degradation in Berlin, Germany, where spring–summer conditions resemble the forward bias phase, and fall–winter conditions resemble the postbias rest phase, thereby validating the effectiveness of the testing procedure as an alternative accelerated aging test.

We used PSCs with a layer stack of glass/ITO/2PACz/perovskite/C_60_/SnO_2_/Cu and a cell area of 0.16 cm^2^ in this study. All PSCs reported in this study were encapsulated by sandwiching cells between two glasses with a butyl edge sealant and polyolefin encapsulant, following the procedure described in our previous study.^33^ The distribution of cell parameters for the encapsulated PSCs reported in this study and a photograph of packaged cells are shown in Figures S1 and S2, respectively. Besides providing excellent shelf life stability (see Figure S3), enabling long-term outdoor tests, and ensuring comparability between samples subjected to indoor and outdoor tests, the encapsulation also excludes the adverse effects of moisture and oxygen during indoor accelerated tests,^34^ which can be exacerbated under an electric field.^35^ The allocation of PSCs to aging tests in this study is shown in Table S1. Note that all solar cell parameters obtained from current–density–voltage (*J*–*V*) measurements presented here were recorded following a 10 min light soaking under open-circuit condition, unless otherwise specified. This preconditioning step is necessary for the consistent history of the cells prior to the *J*–*V* measurements in each of the aging experiments.

To investigate the influence of forward biasing on the cells in the dark, we randomly allocated 22 PSCs from two different batches into three stress levels: 0.4, 0.8, and 1.2 V. A workflow diagram of the three-stress-level aging procedure is shown in Figure S4. Figure 1 shows the evolution of normalized PCE of cells upon aging under these three stress levels. The cells aged at lower stress levels exhibited only a slight decrease in efficiency even after 1750 h of aging. Yet, the performance of the cells aged at 1.2 V rapidly decreased and dropped below the T_80_ line, which marks the time when the PCE reaches 80% of its initial value,^36^ after approximately 130 h of aging on average. After T_80_ was reached, the cells aged at 1.2 V were stored in the dark, referred to as the postbias rest phase, and further characterized during the following period. The evolution of cell parameters for all cells subjected to the test sequence is shown in Figure S5.

**Figure 1:**
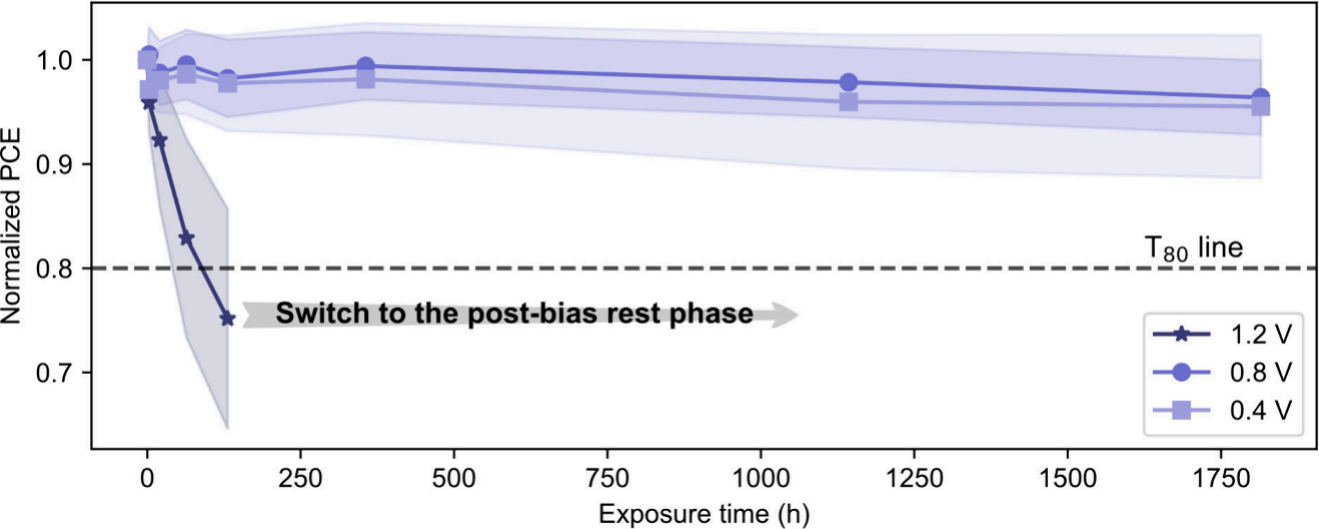
Normalized efficiency evolution of PSCs subjected to different forward bias levels in the dark: 0.4 V (*n* = 6), 0.8 V (*n* = 7), and 1.2 V (*n* = 9). Only PCE values obtained from forward scans are presented. Symbols and lines represent the averages, while the shaded areas indicate the standard deviation. *T*_80_ is represented by the horizontal black dashed line. The gray arrow indicates the start of the postbias phase, which begins once *T*_80_ is reached on average.

The response of a representative cell during the 1.2 V forward bias phase and subsequent postbias rest phase is shown in Figure 2. During forward bias phase (Figure 2a), the short-circuit current (*J*_SC_) and shunt resistance (*R*_SH_) of the cell dramatically decreased, whereas *V*_OC_ stayed steady. During the postbias rest phase (Figure 2b), *J*_SC_ rapidly recovered, and *R*_SH_ showed partial recovery, whereas *V*_OC_ gradually decreased with prolonged postbias rest phase. Note that, at a constant *J*–*V* scan rate maintained throughout the measurements, a slight increase in hysteresis was observed during the forward bias phase. This effect is reversed during the postbias phase (Figure S6). Interestingly, we found that short 10 min light soaking (preconditioning step before measuring the *J*–*V*) had an increasingly detrimental temporary effect on *J*_SC_ and FF. This adverse effect of light soaking completely vanished during the postbias rest phase (Figure S7).

**Figure 2:**
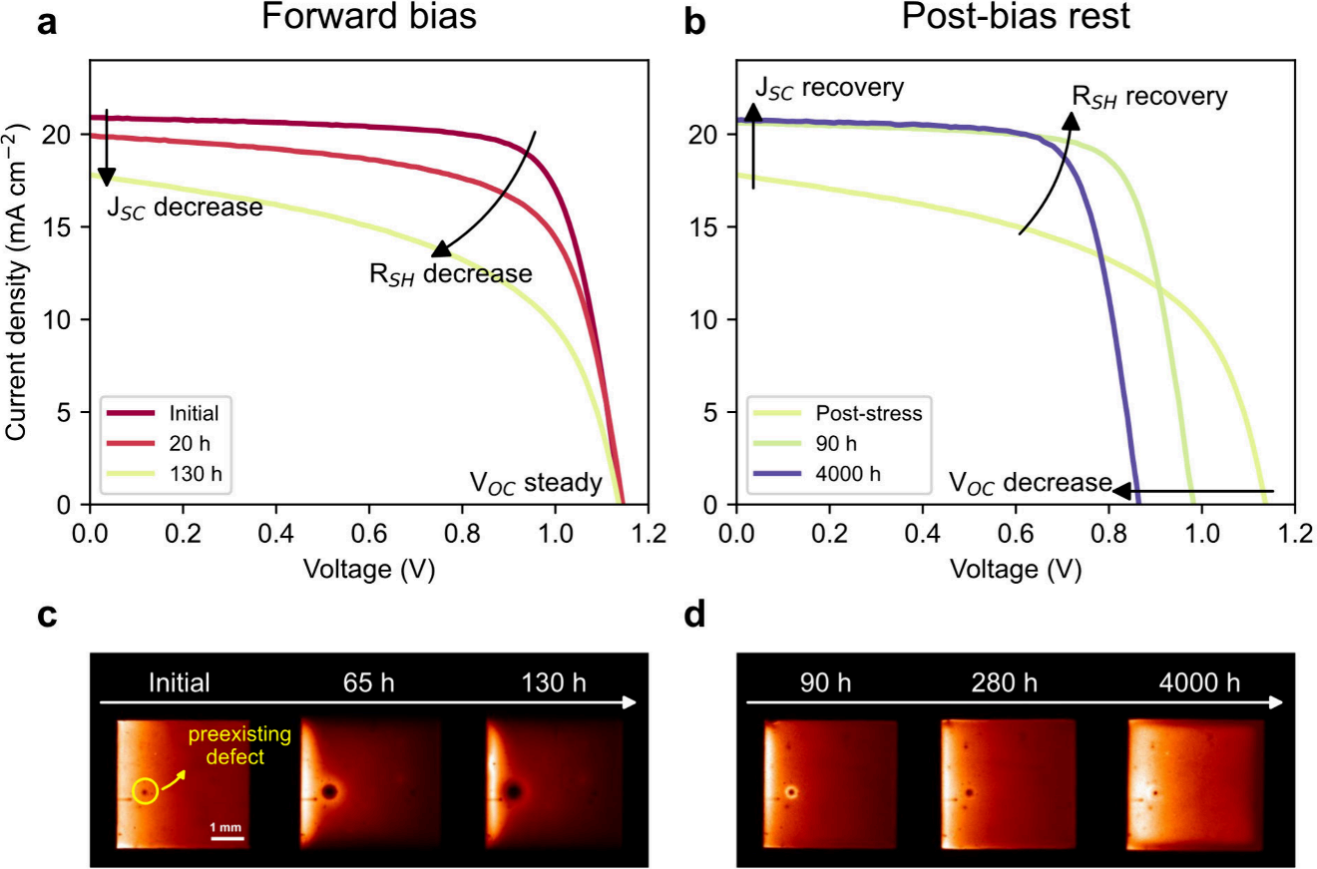
Changes in a representative cell during 1.2 V forward bias (left column) and subsequent postbias rest phases (right column). *J*–*V* curve evolution of the cell during forward bias (a) and postbias rest (b). Only curves from forward scans are shown. See Figure S6 for both scan directions. The 130 h curve from the forward bias phase (a) is reused in (b) as the “poststress” curve to illustrate the starting state for the postbias rest phase. The changes are highlighted with arrows for the most affected cell parameters. Macroscopic changes are revealed by EL images, most notably the growth of a preexisting defect (highlighted with the yellow circle on the initial image) on the cell during the forward bias phase (c) and its return to the initial state during the postbias rest phase (d).

This trend in cell parameters corresponded with changes observed in the electroluminescence (EL) images (Figure 2c,d). Under forward bias, the most notable change was the growth of a preexisting defect (likely manufacturing-related) alongside changes at the cell edges (Figure 2c). These changes returned to the initial state during the postbias rest phase (Figure 2d). Previously, it has been shown that local shunts can cause localized overheating under high reverse bias, leading to perovskite decomposition^37^ or the melting of metal electrode,^19^ ultimately resulting in irreversible degradation. Given the apparently expanded size of the defect in the EL images and tens of hours bias application, such a mechanism would also be expected to result in irreversible degradation in our case. However, the reversibility of changes observed in EL image together with restored charge transport, implies that the mechanism might be driven by a recoverable process. Moreover, similar pre-existing defect growth was observed under continuous illumination of 2.3 suns at open-circuit condition (Figure S8), suggesting current flow is not necessary to drive this effect and a different possible origin will be discussed below. It is important to note that some cells exposed to lower forward bias levels exhibited a similar trend to those observed during 1.2 V forward bias, but to a lesser extent and requiring prolonged exposure (Figure S9). This indicates a potential acceleration of degradation with increasing voltage, although a significantly larger sample size would be required to validate this observation statistically.

As ion migration was considered the primary factor behind the observed changes during 1.2 V forward bias and subsequent postbias rest phases, we implemented the same sequence with shorter exposure durations and conducted in situ optoelectronic characterization at regular intervals to further investigate the role of mobile ions. A workflow diagram of this stress test with in situ characterization (Figure S10) and a table (Table S2) detailing the measurement schedules are shown in the Supporting Information. Figure 3 shows the evolution of three transient measurements of the cell subjected to the test sequence. For a simple overview, the three main states of the cell, initial, at the end of the forward bias phase, and at the end of the postbias phase, are compared in Figure S11. The characteristic slow or low-frequency responses (marked as “ionic signature” in each characterization method in Figure 3) observed in these measurements have been linked to the response of mobile ions in the perovskite,^38−40^ therefore providing insights into the dynamics of mobile ions throughout the test sequence.

**Figure 3:**
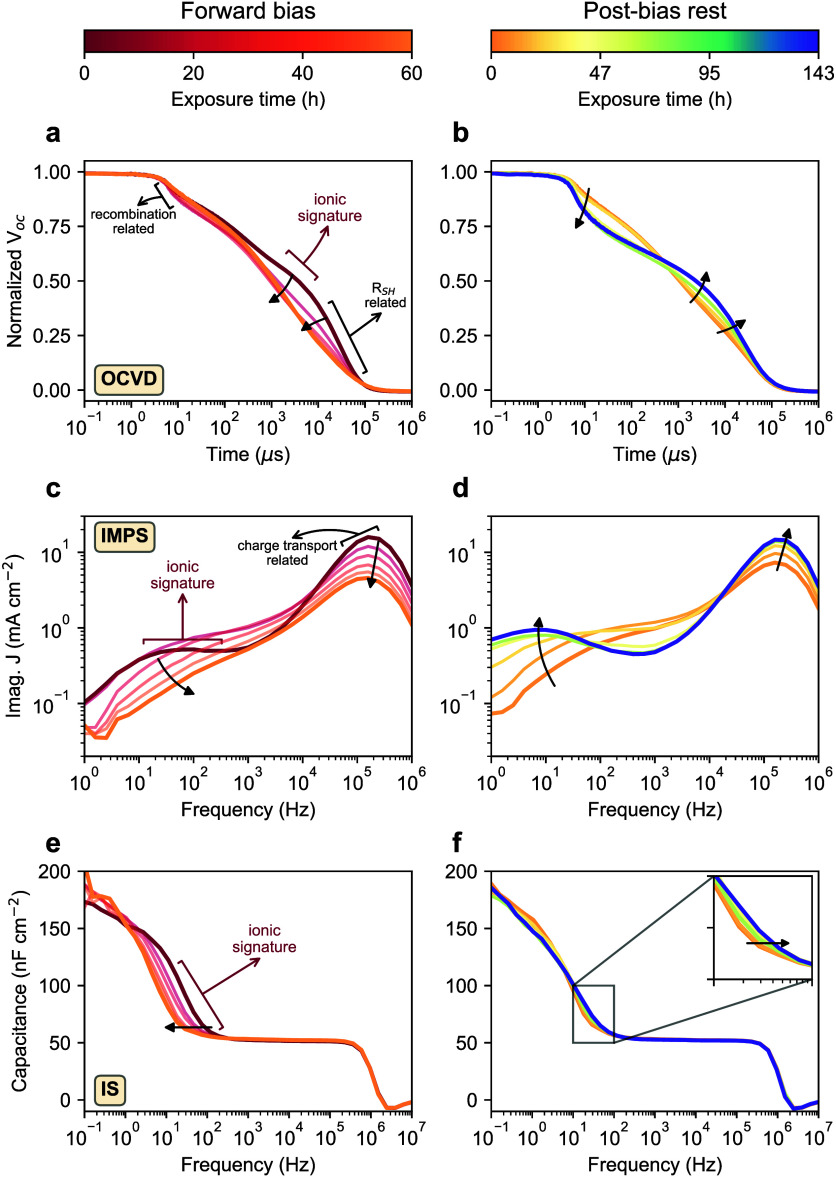
Optoelectronic measurements reveal the dynamics of mobile ions during the 1.2 V forward bias (left column) and postbias rest (right column) phases. (a, b) Normalized open-circuit voltage decay (OCVD). (c, d) Intensity-modulated photocurrent spectroscopy (IMPS). (e, f) Impedance spectroscopy (IS). The inset figure in (f) focuses on the evolution of the transition frequency during the postbias rest phase. The response of the mobile ions, characteristic for each measurement technique, is labeled as “ionic signature” on the initial curve. The exposure time is marked by the given color code.

In open-circuit voltage decay (OCVD), mobile ions in perovskite cause a characteristic shoulder,^38,41^ where the slope of the voltage decay changes.^42^Figure 3a,b show the evolution of normalized OCVD curves in the test sequence (OCVD curves without normalization are provided in Figure S12). During the forward bias phase (Figure 3a), the latest voltage decay, which is governed by *R*_SH_,^43^ occurred more rapidly, indicating a decrease in *R*_SH_. This is consistent with the declining trend in *R*_SH_ shown in Figure 2a. Most intriguingly, the initially observed ionic signature disappeared, resembling the simulated OCVD curves where mobile ions are completely excluded in the perovskite layer.^38^ During the postbias rest phase (Figure 3b), the ionic signature gradually reappeared, and the latest voltage decay recovered, again consistent with the recovery trend in *R*_SH_ in Figure 2b. However, the earliest voltage drop, related to the recombination of free charge carriers near interfaces,^38^ increased. This correlates well with the decrease in *V*_OC_ shown in Figure 2b, which is also evident in the OCVD curves in Figure S12b.

In intensity-modulated photocurrent spectroscopy (IMPS), mobile ions in perovskite lead to a secondary peak at low frequencies.^39,44^Figure 3c,d shows the evolution of the imaginary part of the generated photocurrent over frequency in the test sequence. During the forward bias phase (Figure 3c), the high-frequency peak, related to charge transport,^45^ decreased. More importantly, the initially observed ionic signature at low frequencies gradually disappeared, after which the low-frequency domain corresponded to the simulated IMPS spectra where mobile ions are absent in the perovskite.^39^ During the postbias rest phase (Figure 3d), the high-frequency peak returned to the initial state. The overall trend in the high-frequency peak agrees with the Jsc trend shown in Figure 2a,b. Furthermore, the vanished ionic signature slowly re-emerged and eventually formed a well-defined secondary peak with a higher magnitude and a shift to lower frequency compared to the initial state. This could be caused by changes in mobile ion density or mobility,^39^ traps,^45^ or a combination of these factors during the postbias rest phase.

In impedance spectroscopy (IS), the abrupt increase in capacitance at low frequencies is associated with mobile ionic charges in PSCs.^40,46^Figure 3e,f show the evolution of capacitance over frequency in the test sequence. During the forward bias phase (Figure 3e), the transition frequency gradually shifted to lower frequencies. As the transition frequency is dependent on both ion density and mobility,^39^ this indicates a decrease in either one or both of them. During the postbias rest phase (Figure 3f), the transition frequency shifted back to higher frequencies, but the slope of the capacitance rise changed, resulting in higher capacitance at low frequencies (Figure S11c). This aligns well with IMPS results observed during the postbias rest phase, indicating that the poststress state of the cell is ultimately different from its initial state. Consequently, the signatures of different factors could be intertwined, potentially leading to interfering effects during the postbias phase.

Overall, the ionic signatures initially observed in each of these measurements either disappeared or significantly diminished during the forward bias phase, suggesting that mobile ions became immobilized. During the subsequent postbias rest phase, these ionic signatures either reappeared or evolved into a state different from the initial state, which in turn led to increased recombination, as evidenced by OCVD measurements. In line with the literature, we attribute the whole observation to the dynamic migration of ions. During the forward bias phase, mobile ions migrate to the interfaces due to the created electric field across the absorber.^47^ Additionally, macroscopic manufacturing defects (e.g., contaminations) on perovskite/transport layer interfaces create lateral variations in electrical potential, triggering lateral ion migration.^24^ The lateral ion migration results in the observed growth of the preexisting defect in EL images, consistent with the findings of Jacobs and colleagues.^24^ Consequently, accumulated ions can screen the electric field, thus impeding charge transport,^10,48^ leading to decreases both in *J*_SC_ and *R*_SH_. In this scenario, mobile ions have minimal impact on *V*_OC_;^10^ instead, they can even decrease interfacial recombination rate by reducing the minority carrier accumulation, thus increasing *V*_OC_.^49^ Consequently, *V*_OC_ can remain steady during the forward bias phase (Figures 2a and S7), due to competing effects of ion accumulation and interfacial recombination dynamics.

As mentioned above, we observed that short light soaking triggers a temporary decrease in *J*_SC_ and FF (Figure S7) during the forward bias phase. A comparable phenomenon was earlier observed by Nie et al. in PSCs aged under continuous illumination under open-circuit condition, where both parameters showed recovery after resting in the dark.^25^ They attributed the phenomenon to light-activated metastable trap states, which they propose to originate from the formation of small polarons. Later, Alkhalifah et al. proposed a “defect-polaron” mechanism, in which clustered mobile defects interact with photogenerated charge carriers and lattice, which can lead to light-induced band bending near interfaces.^50^ A similar mechanism might be present in our case, further affecting the charge carrier transport.

During the postbias rest phase, these accumulated ions redistribute, allowing *J*_SC_ and R_SH_ to recover, along with the reversal of observed changes in EL images. Additionally, the adverse effect of light soaking disappears during this phase. However, the redistribution of ions leads to a drastic drop in *V*_OC_. At this stage, OCVD measurements reveal the pronounced initial decay in *V*_OC_ within the 1 to 10 μs regime (Figure 3b) pointing to increased interfacial recombination. During forward bias phase, accumulated ions may initiate degradation at the perovskite/transport layer interfaces,^51,52^ and their redistribution in the postbias rest phase could lead to emergence of detrimental electronic defects.^53,54^ Supporting this hypothesis, the evolution of the *J*–*V* curve during postbias rest phase (Figure 2b) shows good agreement with simulation scenarios, where an injection barrier and significant interfacial recombination are introduced to produce a similar *J*–*V* response.^55^

To provide a structured overview, Table 1 summarizes the features of the degradation process observed in the two phases of the indoor test sequence. These features will be used to assess the relevance of this degradation mechanism under real world operation conditions.

**Table 1:** Summary of Observed Features in the Indoor Test Sequence for Comparison with Outdoor Aging

	forward bias phase	postbias rest phase
EL images	growth of a pre-existing defect	return to initial state
*J*–*V* parameters	*J*_SC_ and *R*_SH_ decrease, *V*_OC_ is steady	*J*_SC_ and *R*_SH_ recover, *V*_OC_ decreases
effect of light soaking	increasingly detrimental to *J*_SC_ and FF	detrimental effect disappears

We conducted outdoor tests in Berlin, Germany, where PSCs were MPP tracked in line with ISOS-O-3 (Figure 4). A workflow diagram of the outdoor aging procedure is shown in Figure S13. We strongly emphasize that multiple stressors acting simultaneously in outdoor environments are ever-changing due to diurnal cycles, weather fluctuations and seasonal variations. This can result in multiple coexisting degradation mechanisms that can be active outdoors. Consequently, achieving a direct alignment between a single indoor test and the outdoor test is extremely challenging, especially when investigating metastable processes caused by ion migration. Nevertheless, the degradation pattern in the outdoor-aged PSCs closely resembles that observed in the indoor test sequence above, as most of the features summarized in Table 1 are present.

**Figure 4:**
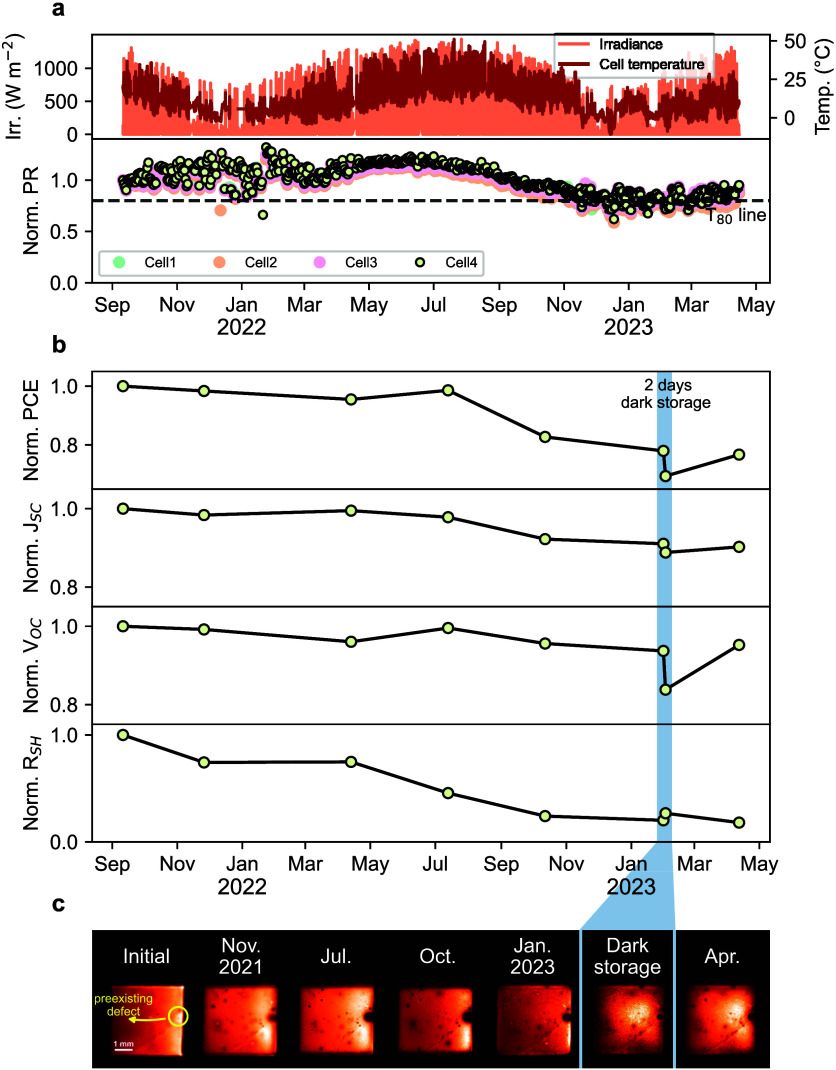
PSCs operated outdoors reveal features that are closely aligned with those observed in the proposed indoor test sequence. (a) Irradiance and cell temperature profiles, along with the normalized PR of four cells, spanning from September 2021 to April 2023. (b) Evolution of cell parameters for a representative cell (Cell4) during outdoor aging, as obtained from indoor *J*–*V* measurements at standard test conditions (1000 W m^–2^, AM 1.5 Spectrum, 25 °C, under sun simulator). Only cell parameters obtained from forward scans are presented. The shaded area depicts 2 days of dark storage indoors. (c) EL images of the cell capturing the evolution of a pre-existing defect (highlighted with the yellow circle on the initial image) during the testing period.

We used performance ratio (PR) to monitor outdoor performance (see Experimental Section, Supporting Information). Figure 4a shows the normalized PR of four cells, along with irradiance and cell temperature profiles, between September 2021 and April 2023. All cells showed an identical trend in outdoor performance. From September to March, the cells exhibited a relatively stable PR, with some variability due to heavy cloud coverage during this period, resulting in more scattered values. This was followed by a clear increase until July, after which the cells experienced a performance drop that continued until stabilizing in the next fall-winter period, with PR beginning to rise again toward April 2023. It is important to note that changes in PR reflect not only cell degradation but also the response to seasonally changing environmental conditions, which is particularly pronounced in PSCs.^29^

These trends in PR are further corroborated by the evolution of *J*–*V* curve parameters (Figure 4b) and EL images (Figure 4c) of a representative cell (Cell4) obtained from indoor measurements, where the features observed in the indoor test sequence become evident. In general, the PCE exhibited a trend similar to the PR. Notably, we observed a decrease in *R*_SH_ along with the distinct growth of a defect from a pre-existing one (Figure 4c) upon outdoor operation. From November to April, *V*_OC_ only slightly decreased. From April to July, *J*_SC_ and *R*_SH_ decreased while *V*_OC_ increased, with the observed defect becoming even more pronounced. After July, all three parameters gradually declined until January, during which the defect began to shrink. At this stage of the outdoor test, the cell was kept indoors in the dark for 2 days (highlighted in blue in Figure 4b,c) to further illustrate the similarity in the degradation mechanism to that observed during the indoor test sequence. Even though *J*_SC_ did not immediately recover, this brief storage period resulted in a rapid shrinking of the defect and a significant drop in *V*_OC_, whereas a slight increase in *R*_SH_, consistent with observations at the postbias rest phase.

Upon returning the cell to outdoor operation, the *V*_OC_ increased again, and the defect started to expand, reversing the changes that occurred during dark storage. Similarly, the cells that went through indoor (bias/rest) test sequence also showed *V*_OC_ increase upon subsequent light soaking under sun simulator (Figure S14). The changes in light soaking dynamics (“fatigue effect”^30,53,56^) during the test sequence is discussed in the Supporting Information, Note 1. Even though light soaking improves the cell’s *V*_OC_ following dark storage, as we demonstrated earlier it becomes increasingly detrimental to the cell’s *J*_SC_ and FF during the forward bias phase, with this adverse effect disappearing upon dark storage. In the outdoor test period shown in Figure 4, we did not observe, or were not able to capture, a significant drop in these cell parameters after 10 min of light soaking in indoor *J*–*V* measurements. However, this feature became evident after extended outdoor aging (Figure S15), where the cell’s *J*_SC_ dropped significantly upon light soaking. After 7 days of dark storage, this adverse effect substantially decreased, similar to the trend in the indoor test sequence.

Overall, we observed all features from the indoor test sequence manifested in the long term outdoor aging in Berlin, Germany. Generally, outdoor operation of the cells during spring–summer period (mean daily irradiance dose ∼5.1 kWh m^–2^, and mean daily maximum cell temperature ∼32 °C) approximates the forward-bias phase, whereas during fall-winter period (mean daily irradiance dose ∼1.6 kWh m^–2^, and mean daily maximum cell temperature ∼14.1 °C), the operation more closely resembles the postbias rest phase, at least during the first 20 months of outdoor tests.

Dark storage following outdoor aging is practical for revealing the features of dynamic ion migration. However, such extended outdoor aging can introduce deviations in cells response to dark storage. In Supporting Information, Note 2, we discuss these deviations and present another cell from a different batch (same device stack and encapsulation) that was subjected to outdoor aging for 25 months and showed almost no deviation from the expected features upon dark storage (Figure S16). This suggests that the dynamics of ion migration also depend on the extent of overall degradation in outdoor-aged cells. The complex interaction between degradation mechanisms highlights the importance of distinguishing recognizable features of each mechanism to trace their presence and contribution to overall efficiency losses. While likely insufficient for accurately forecasting the outdoor lifetime of PSCs, the presented indoor test sequence here is effective at identifying degradation effects due to dynamic ion migration, which undoubtedly contributes to the overall outdoor degradation and leads to peculiar features.

In conclusion, we aged PSCs under prolonged forward bias at three stress levels in the dark and observed rapid degradation when the applied voltage was slightly above cell *V*_OC_ (1.2 V). To capture the complete dynamics of the induced degradation phenomena, we followed the 1.2 V forward bias phase with a postbias rest phase, during which stress is removed, and the cells are stored in the dark. This sequence constitutes the aging procedure proposed in this work. We demonstrated that dynamic ion migration is the dominant mechanism in the test sequence, leading to the deterioration of *J*_SC_ and *R*_SH_, as well as macroscopic defect growth during the forward bias phase. Moreover, a short light soaking causes an additional adverse effect, further deteriorating the cells performance due to the arrangement of ions in the forward bias phase. In the postbias rest phase, these features substantially recover but result in a decrease in *V*_OC_ due to increased recombination. Furthermore, we found that this mechanism plays a key role in outdoor operation in Berlin, Germany, with seasonal variations resembling the forward bias and postbias rest phases in the proposed aging test and leading to similar peculiar features. This indicates that dynamic ion migration is governed not only by diurnal cycles but also by seasonal variations. This insight adds a new dimension to the accelerated aging tests of PSCs, offering a single-stress, no-light approach to simulate dynamic ion migration-induced degradation outdoors. As the mass testing of industrial-size perovskite modules under continuous illumination would be costly, the proposed accelerated aging approach could be an alternative for high throughput, low-cost method to assess module resilience against dynamic ion migration-induced degradation modes.

## Supporting Information Available

Experimental section for perovskite solar cell fabrication, outdoor testing, aging setups, characterization methods. Workflow diagrams of reported stress tests. Additional data for cell parameter distributions, evolution of cell parameters under test sequence, hysteresis behavior, evolution of light soaking behavior during indoor test sequence. A supplementary note on fatigue behavior at indoor tests. A supplementary note on extended outdoor data (PDF)

## References

[ref1] Best Research-Cell Efficiency Chart. https://www.nrel.gov/pv/cell-efficiency.html (accessed 2024–08–25).

[ref2] Bartie N.; Cobos-Becerra L.; Mathies F.; Dagar J.; Unger E.; Fröhling M.; Reuter M. A.; Schlatmann R. Cost versus Environment? Combined Life Cycle, Techno-Economic, and Circularity Assessment of Silicon- and Perovskite-Based Photovoltaic Systems. Journal of Industrial Ecology 2023, 27 (3), 993–1007. 10.1111/jiec.13389.

[ref3] Duan L.; Walter D.; Chang N.; Bullock J.; Kang D.; Phang S. P.; Weber K.; White T.; Macdonald D.; Catchpole K.; Shen H. Stability Challenges for the Commercialization of Perovskite-Silicon Tandem Solar Cells. Nat. Rev. Mater. 2023, 8 (4), 261–281. 10.1038/s41578-022-00521-1.

[ref4] Khenkin M.; Albrecht S. The Way to Predict Outdoor Lifetime. Nat. Energy 2024, 9 (1), 12–13. 10.1038/s41560-023-01419-0.

[ref5] Zhao X.; Liu T.; Burlingame Q. C.; Liu T.; Holley R.; Cheng G.; Yao N.; Gao F.; Loo Y.-L. Accelerated Aging of All-Inorganic, Interface-Stabilized Perovskite Solar Cells. Science 2022, 377 (6603), 307–310. 10.1126/science.abn5679. 35709247

[ref6] Ali M. U.; Mo H.; Li Y.; Djurišić A. B. Outdoor Stability Testing of Perovskite Solar Cells: Necessary Step toward Real-Life Applications. APL Energy 2023, 1 (2), 020903 10.1063/5.0155845.

[ref7] Khenkin M. V.; Katz E. A.; Abate A.; Bardizza G.; Berry J. J.; Brabec C.; Brunetti F.; Bulović V.; Burlingame Q.; Di Carlo A.; Cheacharoen R.; Cheng Y.-B.; Colsmann A.; Cros S.; Domanski K.; Dusza M.; Fell C. J.; Forrest S. R.; Galagan Y.; Di Girolamo D.; Grätzel M.; Hagfeldt A.; von Hauff E.; Hoppe H.; Kettle J.; Köbler H.; Leite M. S.; Liu S.; Loo Y.-L.; Luther J. M.; Ma C.-Q.; Madsen M.; Manceau M.; Matheron M.; McGehee M.; Meitzner R.; Nazeeruddin M. K.; Nogueira A. F.; Odabaşı Ç.; Osherov A.; Park N.-G.; Reese M. O.; De Rossi F.; Saliba M.; Schubert U. S.; Snaith H. J.; Stranks S. D.; Tress W.; Troshin P. A.; Turkovic V.; Veenstra S.; Visoly-Fisher I.; Walsh A.; Watson T.; Xie H.; Yıldırım R.; Zakeeruddin S. M.; Zhu K.; Lira-Cantu M. Consensus Statement for Stability Assessment and Reporting for Perovskite Photovoltaics Based on ISOS Procedures. Nat. Energy 2020, 5 (1), 35–49. 10.1038/s41560-019-0529-5.

[ref8] Boyd C. C.; Cheacharoen R.; Leijtens T.; McGehee M. D. Understanding Degradation Mechanisms and Improving Stability of Perovskite Photovoltaics. Chem. Rev. 2019, 119 (5), 3418–3451. 10.1021/acs.chemrev.8b00336. 30444609

[ref9] Baumann S.; Eperon G. E.; Virtuani A.; Jeangros Q.; Kern D. B.; Barrit D.; Schall J.; Nie W.; Oreski G.; Khenkin M.; Ulbrich C.; Peibst R.; Stein J. S.; Köntges M. Stability and Reliability of Perovskite Containing Solar Cells and Modules: Degradation Mechanisms and Mitigation Strategies. Energy Environ. Sci. 2024, 17 (20), 7566–7599. 10.1039/D4EE01898B.

[ref10] Thiesbrummel J.; Shah S.; Gutierrez-Partida E.; Zu F.; Peña-Camargo F.; Zeiske S.; Diekmann J.; Ye F.; Peters K. P.; Brinkmann K. O.; Caprioglio P.; Dasgupta A.; Seo S.; Adeleye F. A.; Warby J.; Jeangros Q.; Lang F.; Zhang S.; Albrecht S.; Riedl T.; Armin A.; Neher D.; Koch N.; Wu Y.; Le Corre V. M.; Snaith H.; Stolterfoht M. Ion-Induced Field Screening as a Dominant Factor in Perovskite Solar Cell Operational Stability. Nat. Energy 2024, 9 (6), 664–676. 10.1038/s41560-024-01487-w.

[ref11] Motti S. G.; Meggiolaro D.; Barker A. J.; Mosconi E.; Perini C. A. R.; Ball J. M.; Gandini M.; Kim M.; De Angelis F.; Petrozza A. Controlling Competing Photochemical Reactions Stabilizes Perovskite Solar Cells. Nat. Photonics 2019, 13 (8), 532–539. 10.1038/s41566-019-0435-1.

[ref12] Bi E.; Song Z.; Li C.; Wu Z.; Yan Y. Mitigating Ion Migration in Perovskite Solar Cells. Trends in Chemistry 2021, 3 (7), 575–588. 10.1016/j.trechm.2021.04.004.

[ref13] Baishya H.; Adhikari R. D.; Patel M. J.; Yadav D.; Sarmah T.; Alam M.; Kalita M.; Iyer P. K. Defect Mediated Losses and Degradation of Perovskite Solar Cells: Origin, Impacts and Reliable Characterization Techniques. Journal of Energy Chemistry 2024, 94, 217–253. 10.1016/j.jechem.2024.02.062.

[ref14] Hoke E. T.; Slotcavage D. J.; Dohner E. R.; Bowring A. R.; Karunadasa H. I.; McGehee M. D. Reversible Photo-Induced Trap Formation in Mixed-Halide Hybrid Perovskites for Photovoltaics. Chem. Sci. 2015, 6 (1), 613–617. 10.1039/C4SC03141E. 28706629 PMC5491962

[ref15] deQuilettes D. W.; Zhang W.; Burlakov V. M.; Graham D. J.; Leijtens T.; Osherov A.; Bulović V.; Snaith H. J.; Ginger D. S.; Stranks S. D. Photo-Induced Halide Redistribution in Organic-Inorganic Perovskite Films. Nat. Commun. 2016, 7 (1), 11683 10.1038/ncomms11683. 27216703 PMC4890321

[ref16] Kim G. Y.; Senocrate A.; Yang T.-Y.; Gregori G.; Grätzel M.; Maier J. Large Tunable Photoeffect on Ion Conduction in Halide Perovskites and Implications for Photodecomposition. Nat. Mater. 2018, 17 (5), 445–449. 10.1038/s41563-018-0038-0. 29555997

[ref17] Cuzzupè D. T.; Ünlü F.; Lê K.; Bernhardt R.; Wilhelm M.; Grosch M.; Weißing R.; Fischer T.; van Loosdrecht P. H. M.; Mathur S. Thermally-Induced Drift of A-Site Cations at Solid-Solid Interface in Physically Paired Lead Halide Perovskites. Sci. Rep 2022, 12 (1), 10241 10.1038/s41598-022-14452-y. 35715528 PMC9205985

[ref18] Xiao Z.; Yuan Y.; Shao Y.; Wang Q.; Dong Q.; Bi C.; Sharma P.; Gruverman A.; Huang J. Giant Switchable Photovoltaic Effect in Organometal Trihalide Perovskite Devices. Nat. Mater. 2015, 14 (2), 193–198. 10.1038/nmat4150. 25485985

[ref19] Bowring A. R.; Bertoluzzi L.; O’Regan B. C.; McGehee M. D. Reverse Bias Behavior of Halide Perovskite Solar Cells. Adv. Energy Mater. 2018, 8 (8), 1702365 10.1002/aenm.201702365.

[ref20] Eames C.; Frost J. M.; Barnes P. R. F.; O’Regan B. C.; Walsh A.; Islam M. S. Ionic Transport in Hybrid Lead Iodide Perovskite Solar Cells. Nat. Commun. 2015, 6 (1), 7497 10.1038/ncomms8497. 26105623 PMC4491179

[ref21] Bae S.; Kim S.; Lee S.-W.; Cho K. J.; Park S.; Lee S.; Kang Y.; Lee H.-S.; Kim D. Electric-Field-Induced Degradation of Methylammonium Lead Iodide Perovskite Solar Cells. J. Phys. Chem. Lett. 2016, 7 (16), 3091–3096. 10.1021/acs.jpclett.6b01176. 27462013

[ref22] Di Girolamo D.; Phung N.; Kosasih F. U.; Di Giacomo F.; Matteocci F.; Smith J. A.; Flatken M. A.; Köbler H.; Turren Cruz S. H.; Mattoni A.; Cinà L.; Rech B.; Latini A.; Divitini G.; Ducati C.; Di Carlo A.; Dini D.; Abate A. Ion Migration-Induced Amorphization and Phase Segregation as a Degradation Mechanism in Planar Perovskite Solar Cells. Adv. Energy Mater. 2020, 10 (25), 2000310 10.1002/aenm.202000310.

[ref23] Kim M.; Ahn N.; Cheng D.; Xu M.; Ham S.-Y.; Pan X.; Kim S. J.; Luo Y.; Fenning D. P.; Tan D. H. S.; Zhang M.; Zhu G.; Jeong K.; Choi M.; Meng Y. S. Imaging Real-Time Amorphization of Hybrid Perovskite Solar Cells under Electrical Biasing. ACS Energy Lett. 2021, 6 (10), 3530–3537. 10.1021/acsenergylett.1c01707.

[ref24] Jacobs D. A.; Wolff C. M.; Chin X.-Y.; Artuk K.; Ballif C.; Jeangros Q. Lateral Ion Migration Accelerates Degradation in Halide Perovskite Devices. Energy Environ. Sci. 2022, 15 (12), 5324–5339. 10.1039/D2EE02330J.

[ref25] Nie W.; Blancon J.-C.; Neukirch A. J.; Appavoo K.; Tsai H.; Chhowalla M.; Alam M. A.; Sfeir M. Y.; Katan C.; Even J.; Tretiak S.; Crochet J. J.; Gupta G.; Mohite A. D. Light-Activated Photocurrent Degradation and Self-Healing in Perovskite Solar Cells. Nat. Commun. 2016, 7 (1), 11574 10.1038/ncomms11574. 27181192 PMC4873646

[ref26] Prete M.; Khenkin M. V.; Glowienka D.; Patil B. R.; Lissau J. S.; Dogan I.; Hansen J. L.; Leißner T.; Fiutowski J.; Rubahn H.-G.; Julsgaard B.; Balling P.; Turkovic V.; Galagan Y.; Katz E. A.; Madsen M. Bias-Dependent Dynamics of Degradation and Recovery in Perovskite Solar Cells. ACS Appl. Energy Mater. 2021, 4 (7), 6562–6573. 10.1021/acsaem.1c00588.

[ref27] Mamun A. A.; Ava T. T.; Byun H. R.; Jeong H. J.; Jeong M. S.; Nguyen L.; Gausin C.; Namkoong G. Unveiling the Irreversible Performance Degradation of Organo-Inorganic Halide Perovskite Films and Solar Cells during Heating and Cooling Processes. Phys. Chem. Chem. Phys. 2017, 19 (29), 19487–19495. 10.1039/C7CP03106H. 28718472

[ref28] Singh R.; Hu H.; Feeney T.; Diercks A.; Laufer F.; Li Y.; Duong T.; Schackmar F.; Nejand B. A.; Paetzold U. W. Danger in the Dark: Stability of Perovskite Solar Cells with Varied Stoichiometries and Morphologies Stressed at Various Conditions. ACS Appl. Mater. Interfaces 2024, 16 (21), 27450–27462. 10.1021/acsami.4c04350. 38751205

[ref29] Khenkin M.; Köbler H.; Remec M.; Roy R.; Erdil U.; Li J.; Phung N.; Adwan G.; Paramasivam G.; Emery Q.; Unger E.; Schlatmann R.; Ulbrich C.; Abate A. Light Cycling as a Key to Understanding the Outdoor Behaviour of Perovskite Solar Cells. Energy Environ. Sci. 2024, 17 (2), 602–610. 10.1039/D3EE03508E.

[ref30] Huang F.; Jiang L.; Pascoe A. R.; Yan Y.; Bach U.; Spiccia L.; Cheng Y.-B. Fatigue Behavior of Planar CH3NH3PbI3 Perovskite Solar Cells Revealed by Light on/off Diurnal Cycling. Nano Energy 2016, 27, 509–514. 10.1016/j.nanoen.2016.07.033.

[ref31] Domanski K.; Roose B.; Matsui T.; Saliba M.; Turren-Cruz S.-H.; Correa-Baena J.-P.; Carmona C. R.; Richardson G.; Foster J. M.; De Angelis F.; Ball J. M.; Petrozza A.; Mine N.; Nazeeruddin M. K.; Tress W.; Grätzel M.; Steiner U.; Hagfeldt A.; Abate A. Migration of Cations Induces Reversible Performance Losses over Day/Night Cycling in Perovskite Solar Cells. Energy Environ. Sci. 2017, 10 (2), 604–613. 10.1039/C6EE03352K.

[ref32] Khenkin M. V.; K. M A.; Visoly-Fisher I.; Kolusheva S.; Galagan Y.; Di Giacomo F.; Vukovic O.; Patil B. R.; Sherafatipour G.; Turkovic V.; Rubahn H.-G.; Madsen M.; Mazanik A. V.; Katz E. A. Dynamics of Photoinduced Degradation of Perovskite Photovoltaics: From Reversible to Irreversible Processes. ACS Appl. Energy Mater. 2018, 1 (2), 799–806. 10.1021/acsaem.7b00256.

[ref33] Emery Q.; Remec M.; Paramasivam G.; Janke S.; Dagar J.; Ulbrich C.; Schlatmann R.; Stannowski B.; Unger E.; Khenkin M. Encapsulation and Outdoor Testing of Perovskite Solar Cells: Comparing Industrially Relevant Process with a Simplified Lab Procedure. ACS Appl. Mater. Interfaces 2022, 14 (4), 5159–5167. 10.1021/acsami.1c14720. 35108814

[ref34] Chu Q.-Q.; Sun Z.; Wang D.; Cheng B.; Wang H.; Wong C.-P.; Fang B. Encapsulation: The Path to Commercialization of Stable Perovskite Solar Cells. Matter 2023, 6 (11), 3838–3863. 10.1016/j.matt.2023.08.016.

[ref35] Barbé J.; Kumar V.; Newman M. J.; Lee H. K. H.; Jain S. M.; Chen H.; Charbonneau C.; Rodenburg C.; Tsoi W. C. Dark Electrical Bias Effects on Moisture-Induced Degradation in Inverted Lead Halide Perovskite Solar Cells Measured by Using Advanced Chemical Probes. Sustainable Energy Fuels 2018, 2 (4), 905–914. 10.1039/C7SE00545H.

[ref36] Reese M. O.; Gevorgyan S. A.; Jørgensen M.; Bundgaard E.; Kurtz S. R.; Ginley D. S.; Olson D. C.; Lloyd M. T.; Morvillo P.; Katz E. A.; Elschner A.; Haillant O.; Currier T. R.; Shrotriya V.; Hermenau M.; Riede M.; R. Kirov K.; Trimmel G.; Rath T.; Inganäs O.; Zhang F.; Andersson M.; Tvingstedt K.; Lira-Cantu M.; Laird D.; McGuiness C.; Gowrisanker S.; Pannone M.; Xiao M.; Hauch J.; Steim R.; DeLongchamp D. M.; Rösch R.; Hoppe H.; Espinosa N.; Urbina A.; Yaman-Uzunoglu G.; Bonekamp J.-B.; van Breemen A. J. J. M.; Girotto C.; Voroshazi E.; Krebs F. C. Consensus Stability Testing Protocols for Organic Photovoltaic Materials and Devices. Sol. Energy Mater. Sol. Cells 2011, 95 (5), 1253–1267. 10.1016/j.solmat.2011.01.036.

[ref37] Bogachuk D.; Saddedine K.; Martineau D.; Narbey S.; Verma A.; Gebhardt P.; Herterich J. P.; Glissmann N.; Zouhair S.; Markert J.; Gould I. E.; McGehee M. D.; Würfel U.; Hinsch A.; Wagner L. Perovskite Photovoltaic Devices with Carbon-Based Electrodes Withstanding Reverse-Bias Voltages up to −9 V and Surpassing IEC 61215:2016 International Standard. Solar RRL 2022, 6 (3), 2100527 10.1002/solr.202100527.

[ref38] Fischer M.; Kiermasch D.; Gil-Escrig L.; Bolink H. J.; Dyakonov V.; Tvingstedt K. Assigning Ionic Properties in Perovskite Solar Cells; a Unifying Transient Simulation/Experimental Study. Sustainable Energy Fuels 2021, 5 (14), 3578–3587. 10.1039/D1SE00369K.

[ref39] Neukom M. T.; Schiller A.; Züfle S.; Knapp E.; Ávila J.; Pérez-del-Rey D.; Dreessen C.; Zanoni K. P. S.; Sessolo M.; Bolink H. J.; Ruhstaller B. Consistent Device Simulation Model Describing Perovskite Solar Cells in Steady-State, Transient, and Frequency Domain. ACS Appl. Mater. Interfaces 2019, 11 (26), 23320–23328. 10.1021/acsami.9b04991. 31180209

[ref40] Futscher M. H.; Gangishetty M. K.; Congreve D. N.; Ehrler B. Quantifying Mobile Ions and Electronic Defects in Perovskite-Based Devices with Temperature-Dependent Capacitance Measurements: Frequency vs Time Domain. J. Chem. Phys. 2020, 152 (4), 044202 10.1063/1.5132754. 32007073

[ref41] Vidani A. C.; Jenatsch S.; Kothandraman R.; Fu F.; Gadola A.; Zuefle S.; Ruhstaller B. Aging and Characterization of High-Bandgap Perovskites for All Thin-Film Tandem Solar Cell Devices. In Organic, Hybrid, and Perovskite Photovoltaics XXIV; SPIE, 2023; Vol. 12660, pp 18–26. 10.1117/12.2676914.

[ref42] Pockett A.; Eperon G. E.; Sakai N.; Snaith H. J.; Peter L. M.; Cameron P. J. Microseconds, Milliseconds and Seconds: Deconvoluting the Dynamic Behaviour of Planar Perovskite Solar Cells. Phys. Chem. Chem. Phys. 2017, 19 (8), 5959–5970. 10.1039/C6CP08424A. 28177002

[ref43] Lemaire A.; Perona A.; Caussanel M.; Duval H.; Dollet A. Open-Circuit Voltage Decay: Moving to a Flexible Method of Characterisation. IET Circuits, Devices & Systems 2020, 14 (7), 947–955. 10.1049/iet-cds.2020.0123.

[ref44] Correa-Baena J.; Anaya M.; Lozano G.; Tress W.; Domanski K.; Saliba M.; Matsui T.; Jacobsson T. J.; Calvo M. E.; Abate A.; Grätzel M.; Míguez H.; Hagfeldt A. Unbroken Perovskite: Interplay of Morphology, Electro-optical Properties, and Ionic Movement. Adv. Mater. 2016, 28 (25), 5031–5037. 10.1002/adma.201600624. 27122472

[ref45] Neukom M.; Züfle S.; Jenatsch S.; Ruhstaller B. Opto-Electronic Characterization of Third-Generation Solar Cells. Sci. Technol. Adv. Mater. 2018, 19 (1), 291–316. 10.1080/14686996.2018.1442091. 29707069 PMC5917438

[ref46] Mohammadian N.; Moshaii A.; Alizadeh A.; Gharibzadeh S.; Mohammadpour R. Influence of Perovskite Morphology on Slow and Fast Charge Transport and Hysteresis in the Perovskite Solar Cells. J. Phys. Chem. Lett. 2016, 7 (22), 4614–4621. 10.1021/acs.jpclett.6b01909. 27804296

[ref47] Zuo L.; Li Z.; Chen H. Ion Migration and Accumulation in Halide Perovskite Solar Cells. Chin. J. Chem. 2023, 41 (7), 861–876. 10.1002/cjoc.202200505.

[ref48] Le Corre V. M.; Diekmann J.; Peña-Camargo F.; Thiesbrummel J.; Tokmoldin N.; Gutierrez-Partida E.; Peters K. P.; Perdigón-Toro L.; Futscher M. H.; Lang F.; Warby J.; Snaith H. J.; Neher D.; Stolterfoht M. Quantification of Efficiency Losses Due to Mobile Ions in Perovskite Solar Cells via Fast Hysteresis Measurements. Solar RRL 2022, 6 (4), 2100772 10.1002/solr.202100772.

[ref49] Hart L. J. F.; Angus F. J.; Li Y.; Khaleed A.; Calado P.; Durrant J. R.; Djurisic A. B.; Docampo P.; Barnes P. R. F. More Is Different: Mobile Ions Improve the Design Tolerances of Perovskite Solar Cells. Energy Environ. Sci. 2024, 17 (19), 7107–7118. 10.1039/D4EE02669A.

[ref50] Alkhalifah G.; Marshall A. D.; Rudayni F.; Wanigasekara S.; Wu J. Z.; Chan W.-L. Defect-Polaron and Enormous Light-Induced Fermi-Level Shift at Halide Perovskite Surface. J. Phys. Chem. Lett. 2022, 13 (29), 6711–6720. 10.1021/acs.jpclett.2c01940. 35849072

[ref51] Ni Z.; Jiao H.; Fei C.; Gu H.; Xu S.; Yu Z.; Yang G.; Deng Y.; Jiang Q.; Liu Y.; Yan Y.; Huang J. Evolution of Defects during the Degradation of Metal Halide Perovskite Solar Cells under Reverse Bias and Illumination. Nat. Energy 2022, 7 (1), 65–73. 10.1038/s41560-021-00949-9.

[ref52] Sakhatskyi K.; John R. A.; Guerrero A.; Tsarev S.; Sabisch S.; Das T.; Matt G. J.; Yakunin S.; Cherniukh I.; Kotyrba M.; Berezovska Y.; Bodnarchuk M. I.; Chakraborty S.; Bisquert J.; Kovalenko M. V. Assessing the Drawbacks and Benefits of Ion Migration in Lead Halide Perovskites. ACS Energy Lett. 2022, 7 (10), 3401–3414. 10.1021/acsenergylett.2c01663. 36277137 PMC9578653

[ref53] Jiang L.; Lu J.; Raga S. R.; Sun J.; Lin X.; Huang W.; Huang F.; Bach U.; Cheng Y.-B. Fatigue Stability of CH3NH3PbI3 Based Perovskite Solar Cells in Day/Night Cycling. Nano Energy 2019, 58, 687–694. 10.1016/j.nanoen.2019.02.005.

[ref54] Wang J.; Duan X.; Yin W.-J. Photoinduced Dynamic Defects Responsible for the Giant, Reversible, and Bidirectional Light-Soaking Effect in Perovskite Solar Cells. J. Phys. Chem. Lett. 2021, 12 (38), 9328–9335. 10.1021/acs.jpclett.1c02929. 34546066

[ref55] These A.; Koster L. J. A.; Brabec C. J.; Le Corre V. M. Beginner’s Guide to Visual Analysis of Perovskite and Organic Solar Cell Current Density-Voltage Characteristics. Adv. Energy Mater. 2024, 14 (21), 2400055 10.1002/aenm.202400055.

[ref56] Zhang Y.; Song Q.; Liu G.; Chen Y.; Guo Z.; Li N.; Niu X.; Qiu Z.; Zhou W.; Huang Z.; Zhu C.; Zai H.; Ma S.; Bai Y.; Chen Q.; Huang W.; Zhao Q.; Zhou H. Improved Fatigue Behaviour of Perovskite Solar Cells with an Interfacial Starch-Polyiodide Buffer Layer. Nat. Photonics 2023, 17 (12), 1066–1073. 10.1038/s41566-023-01287-w.

