# Supplementary material for: Mimicking Outdoor Ion Migration in Perovskite Solar Cells: A Forward Bias, No-Light Accelerated Aging Approach
Source: ACS Energy Lett. 2025 Mar 5;10(3):1529–37. doi: 10.1021/acsenergylett.5c00376 (PMC11915753; doi:10.1021/acsenergylett.5c00376)
Supplement: Supplementary file 1 — nz5c00376_si_001.pdf [file nz5c00376_si_001.pdf]

Supporting Information

Mimicking Outdoor Ion Migration in Perovskite Solar Cells: A Forward Bias, No-Light Accelerated Aging Approach

Ulas Erdil[1,2], Mark Khenkin[1], Marko Remec[1,3], Quiterie Emery[1], Vediappan Sudhakar[4a], Rutger Schlatmann[1,5], Antonio Abate[1,2], Eugene A. Katz[4], Carolin Ulbrich[1]*

[1]Helmholtz-Zentrum Berlin für Materialien und Energie, Hahn-Meitner-Platz 1, 14109 Berlin, Germany

[2]Faculty of Chemistry, Bielefeld University, 33615 Bielefeld, Germany

[3]Faculty of Electrical Engineering, University of Ljubljana, 1000 Ljubljana, Slovenia

[4]Ben-Gurion National Solar Energy Center, Swiss Institute for Dryland Environmental and Energy Research, Jacob Blaustein Institutes for Desert Research, Ben-Gurion University of the Negev, Midreshet Ben-Gurion 84990, Israel

[5]Faculty 1 – Energy and Information, Hochschule für Technik und Wirtschaft Berlin, 10313 Berlin, Germany

Present address:

[a] Université Grenoble Alpes, CEA, CNRS, IRIG/SyMMES/STEP, 38000 Grenoble, France.

Corresponding Author

*Corresponding author: mark.khenkin@helmholtz-berlin.de

Experimental Section

Solar cell fabrication: Perovskite solar cells with a stack of glass/ITO/2PACz/ $Cs_{0.17}FA_{0.83}PbI_{2.49}Br_{0.51}$ /C_{60}/SnO_2/Cu were fabricated following the procedure described in prior work[1].

Outdoor test: The outdoor test was conducted in Berlin, Germany (52°25'53.5"N, 13°31'27.7"E) on the rooftop setup detailed in previous work[2]. The performance ratio (PR) was calculated daily from 6 a.m. to 8 p.m. using the following formula:

$$PR = \frac{\int_{06:00}^{20:00} P_{MPP}\ (t)\ dt}{\int_{06:00}^{20:00} P_{irr}\ (t)\ dt}$$

where P_{MPP} is the power output at maximum power point while P_{irr} is the incident irradiance on the cells. For normalized PR, daily PR values were normalized to the PR on the first full day of operation (the next day following the installation). Finally, a Hampel filter[3] was applied to the data to exclude outliers, such as days when the cells were taken indoors for measurements. For a quantitative definition of the seasonal periods, the mean daily irradiance dose and mean daily maximum cell temperature were calculated for the timeframe between September 2021 and September 2022.

Three-stress level aging test: A custom-made setup consisting of three separate power supplies (VOLTCRAFT, LPS 1153) were employed to apply three different voltages. The electrical connections were routed into an enclosed box where PSCs were housed to ensure cells remained in complete darkness throughout the stress test.

Aging test with in-situ characterization: The aging test with in-situ characterization was conducted using the PAIOS setup (Fluxim AG). The PCSs were placed in an enclosed, light-tight structure to ensure complete darkness. A constant voltage of 1.2 V was applied to the connected solar cell for specific intervals. After each stress interval, the applied voltage was halted, followed by a 10-minute resting time. Following the resting period, a 10-minute light soaking was applied using the setup's white light emitting diode (LED), and then the in-situ optoelectronic characterization routine was carried out. For the sequential post-bias rest phase, the same procedure was applied, but without any voltage being applied during predefined intervals.

EL measurement: EL images were captured using a LumiSolarMobile System from GreatEyes, equipped with a charge-coupled device (CCD) camera. The power for the measurements was supplied by a programmable power supply (GEN1U1500W, TDK). A current density of 25 mA cm^{-2} (slightly above J_{SC}) was applied. The integration time was varied to accumulate significant signals, especially for degraded samples. Images shown in Figure 2c-d, Figure 4c and Figure S3 c-d were scaled to the maximum intensity of each image, therefore, the absolute scales could differ from image to image.

J-V measurement: J-V measurements were performed under simulated AM1.5G spectrum at 1 sun illumination using a calibrated dual-source solar simulator (WXS-155S-L, class AAA, Wacom) and a Keithley 238 SMU. The substrates were placed on a temperature-controlled chuck maintained at 25 °C. The voltage was swept first in the forward direction (from -0.2 to 1.2 V) and then in the reverse direction (from 1.2 to -0.2 V), with 0.01 V steps. The integration time was 20 ms, and the settling time was 20 ms.

In-situ optoelectronic characterization: The in-situ characterization shown in Figure 3 was performed using PAIOS setup. The measurements were conducted several times after each predefined interval in the aging test sequence. For those measurements that require illumination, a white LED was the illumination source. In *open-circuit voltage decay (OCVD),* the light pulse length was set to 1 second without initial settling time or offset light intensity. *Intensity modulated photocurrent spectroscopy (IMPS)* measurements were performed at short-circuit condition with zero offset voltage, covering a frequency range from 1 MHz to 1 Hz. The light intensity was modulated with a 90% offset and a 10% amplitude. *Impedance spectroscopy (IS)* measurements were performed without illumination (dark) at zero offset voltage with an amplitude of 70 mV, covering a frequency range from 10 MHz to 100 mHz.

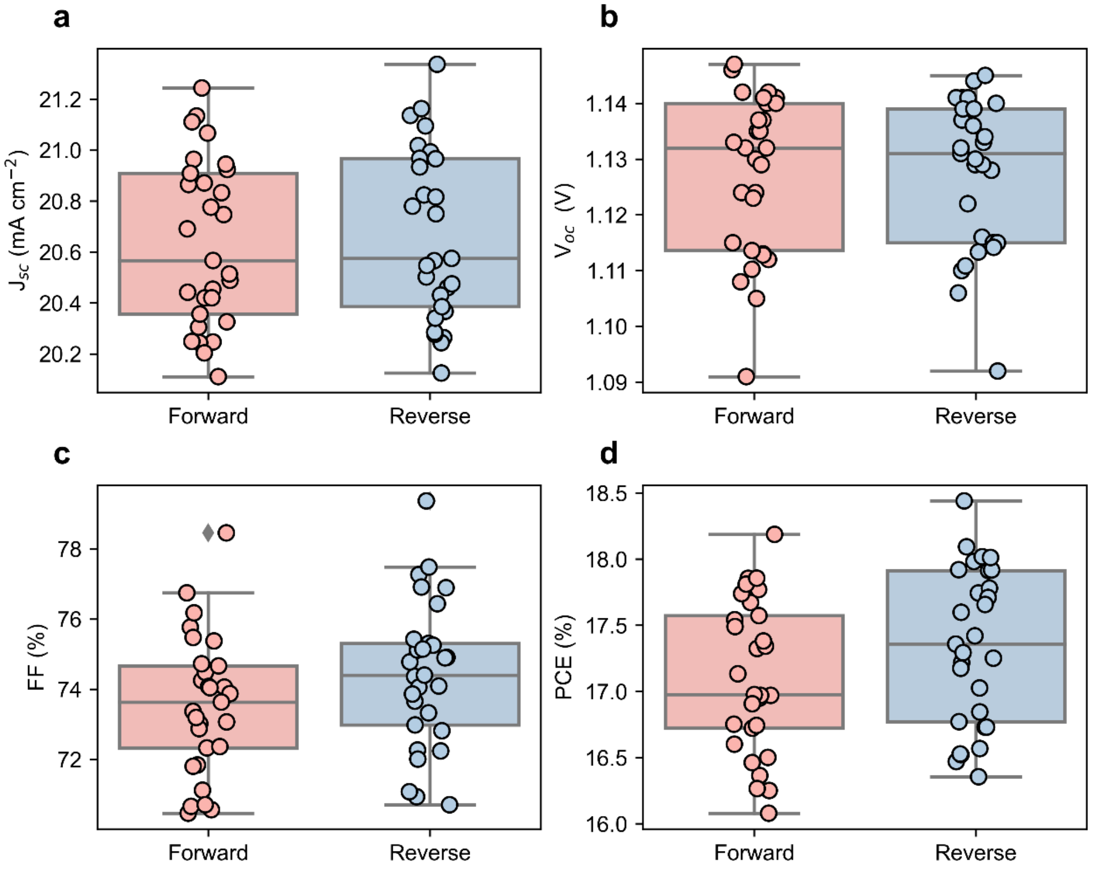

Figure S1. Distribution of cell parameters of the encapsulated PSCs reported in this study.

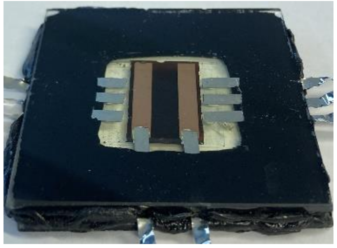

Figure S2. Photograph of an encapsulated substrate containing six electrically isolated PSCs.

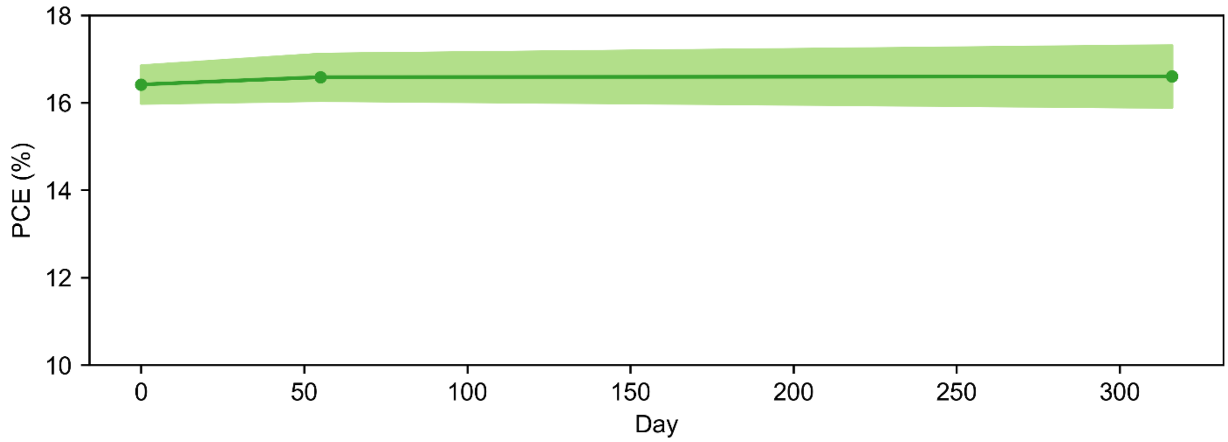

Figure S3. The shelf-life stability of encapsulated PSCs (n =3) under ambient conditions. Symbol and line represent the average, while filled area indicate standard deviation.

No. of substrates allocated to the test	No. of cells tested on the substrate(s)	Test as mentioned in the text	Test specification
1	4	Outdoor	ISOS-O-3, Berlin, Germany
2	6	Three stress level aging	0.4 V
2	7	Three stress level aging	0.8 V
2	9	Three stress level aging	1.2 V + dark storage
1	1	Stress test with in-situ characterization	1.2 V + dark storage
1	3	Shelf-life	Shelf storage

Table S1. Allocation of substrates(cells) to the aging tests. Each substrate(cells) was exposed to a dedicated test without undergoing prior stress and was not restressed after the test was completed.

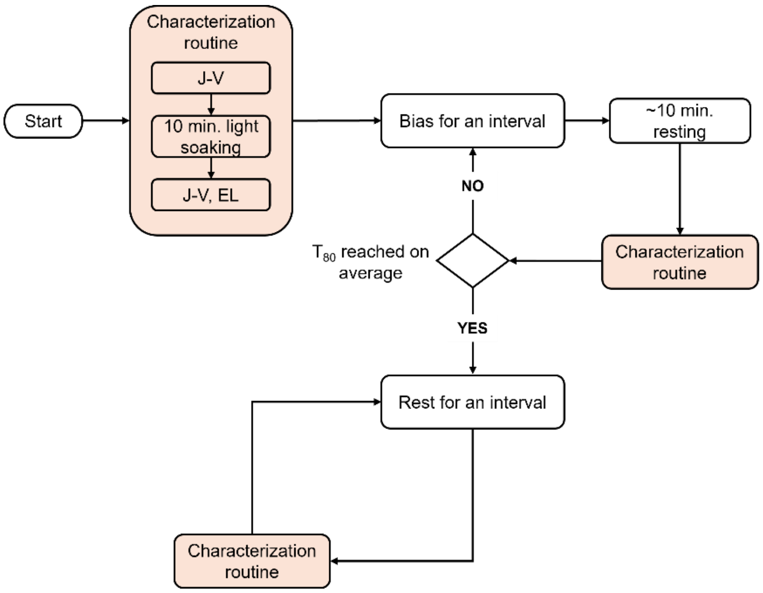

Figure S4. Workflow diagram illustrating the three-stress-level aging test procedure.

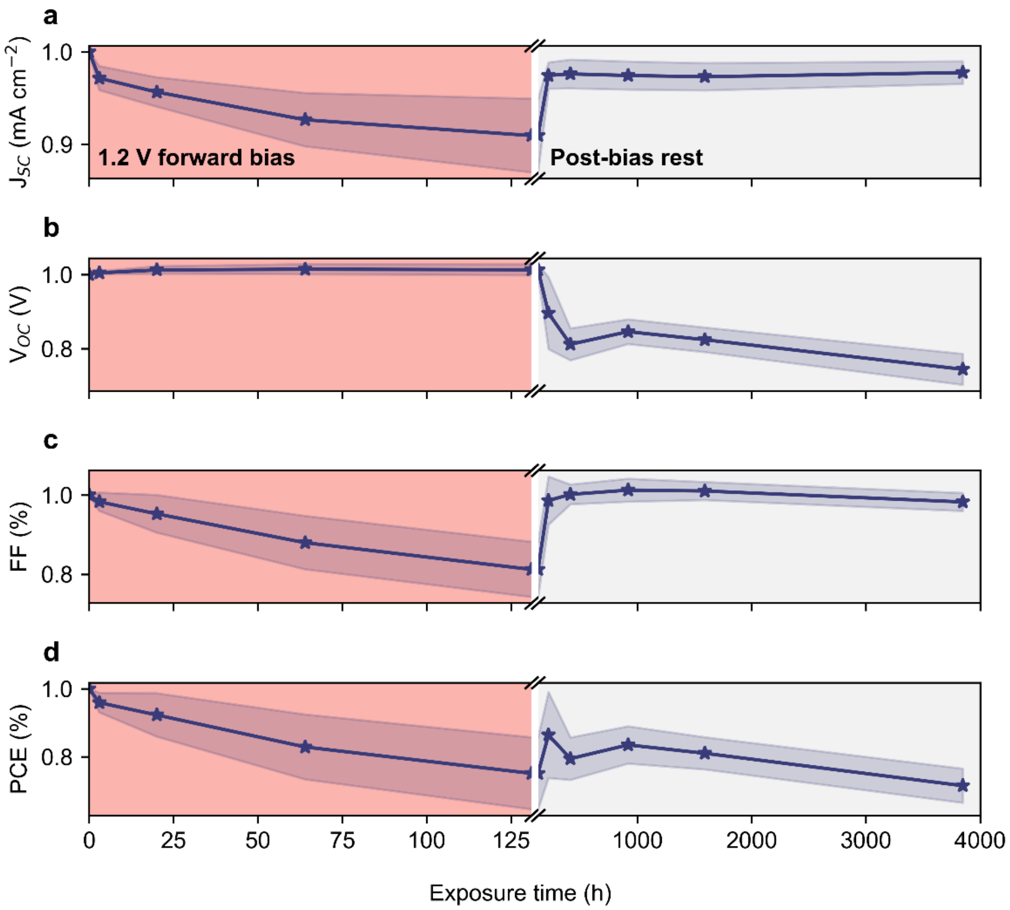

Figure S5. Evolution of main cell parameters (normalized) during the indoor test sequence, for all cells (n=9) subjected to 1.2 V forward bias and subsequent post-bias phase. **(a)** J_{SC}, **(b)** V_{OC}, **(c)** FF and **(d)** PCE. The red shaded background represents the forward bias phase, while the grey shaded background marks the post-bias rest phase. All parameters are normalized to their initial values.

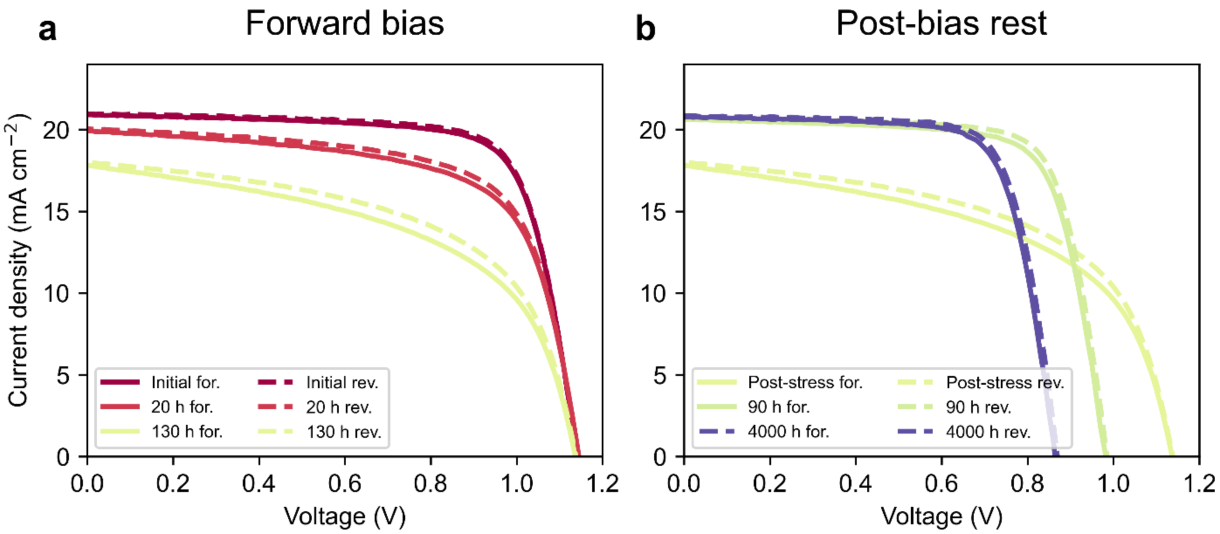

Figure S6. At a constant scan rate, hysteresis slightly increases during the bias phase and recovers in the post-bias rest phase.

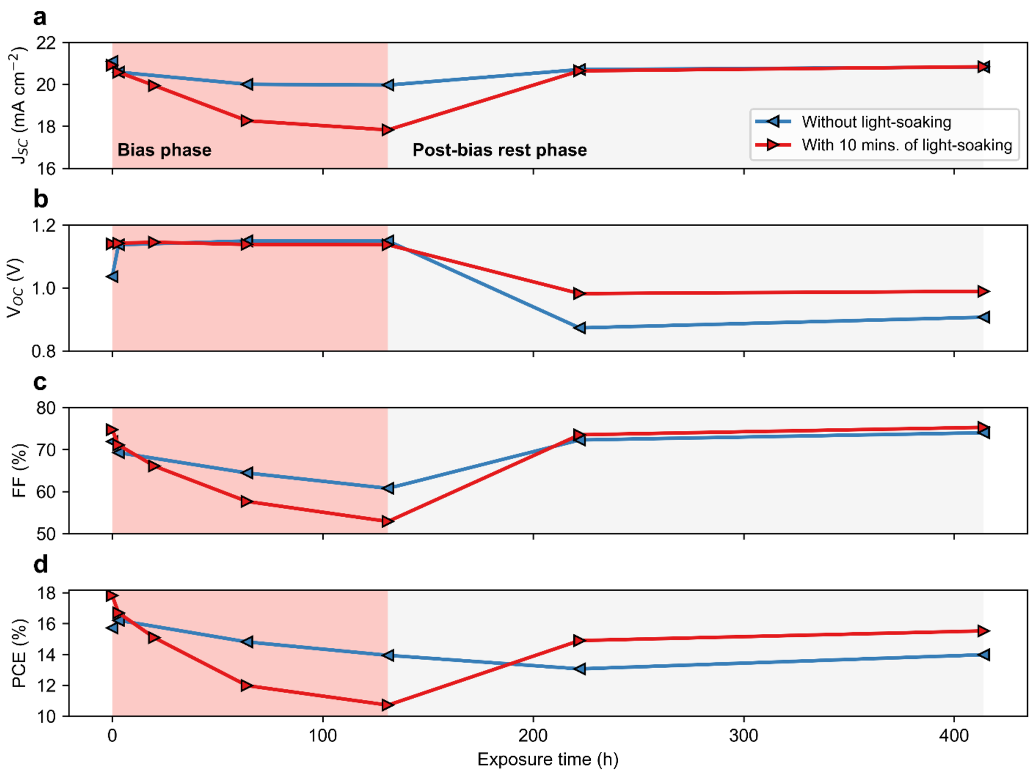

Figure S7. Short light soaking reveals an additional adverse effect that further decreases Jsc and FF, but the effect disappears during the post-bias rest phase.

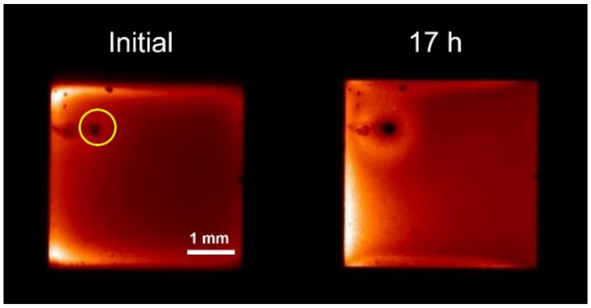

Figure S8. Preexisting defect growth under constant illumination (2.3 suns) at open-circuit conditions. The yellow circle on the initial EL image highlights the preexisting defect, which later grows under constant illumination at open-circuit conditions.

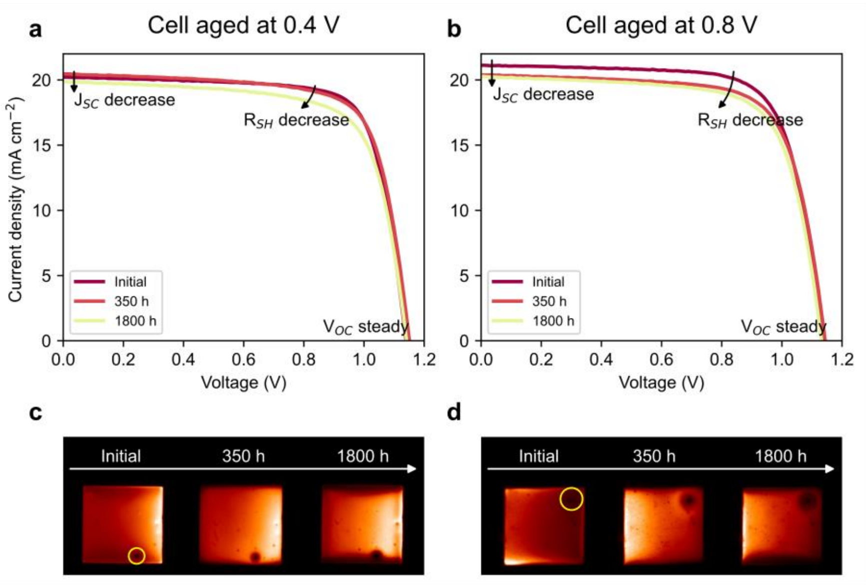

Figure S9. Some cells subjected to lower bias stresses (0.4 and 0.8 V) exhibit the same features as those observed at 1.2 V, but with significantly prolonged exposure to stress. **(a-c)** Changes in J-V curves and EL images for a cell subjected to 0.4 V, and **(b-e)** for a cell subjected to 0.8 V forward bias in the dark. Yellow circles on the initial EL images highlight the preexisting defect, which later grows.

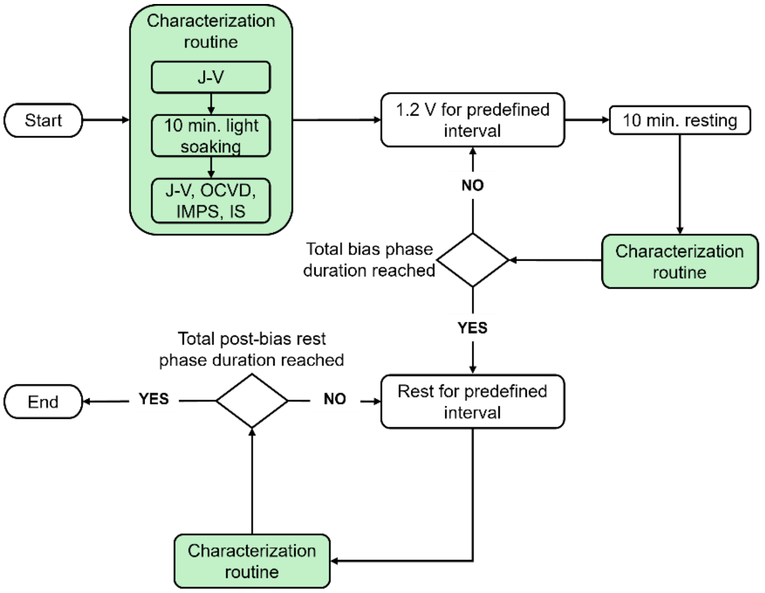

Figure S10. Workflow diagram illustrating the test sequence with in-situ optoelectronic characterization.

Test phase	N^{th} measurement	Measured at (h)	Total test duration (h)
Bias phase	1	0	0
	2	3	3
	3	6	6
	4	12	12
	5	24	24
	6	36	36
	7	48	48
	8	60	60
Post-bias rest phase	9	6	66
	10	12	72
	11	24	84
	12	48	108
	13	70	130
	14	143	203

Table S2. Measurement schedule during the test sequence with in-situ optoelectronic characterization. The second and third measurements are not presented in Figure 3 to improve the readability.

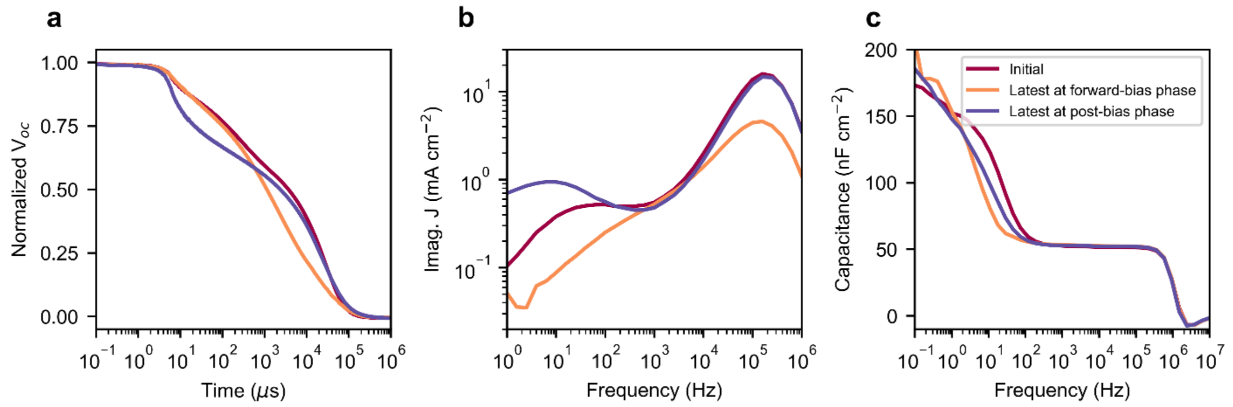

Figure S11. Overview of the three main states of the cell state during the test sequence: initial, latest at at forward-bias phase and latest at post-bias rest phase for **(a)** normalized OCVD, **(b)** IMPS and **(c)** IS.

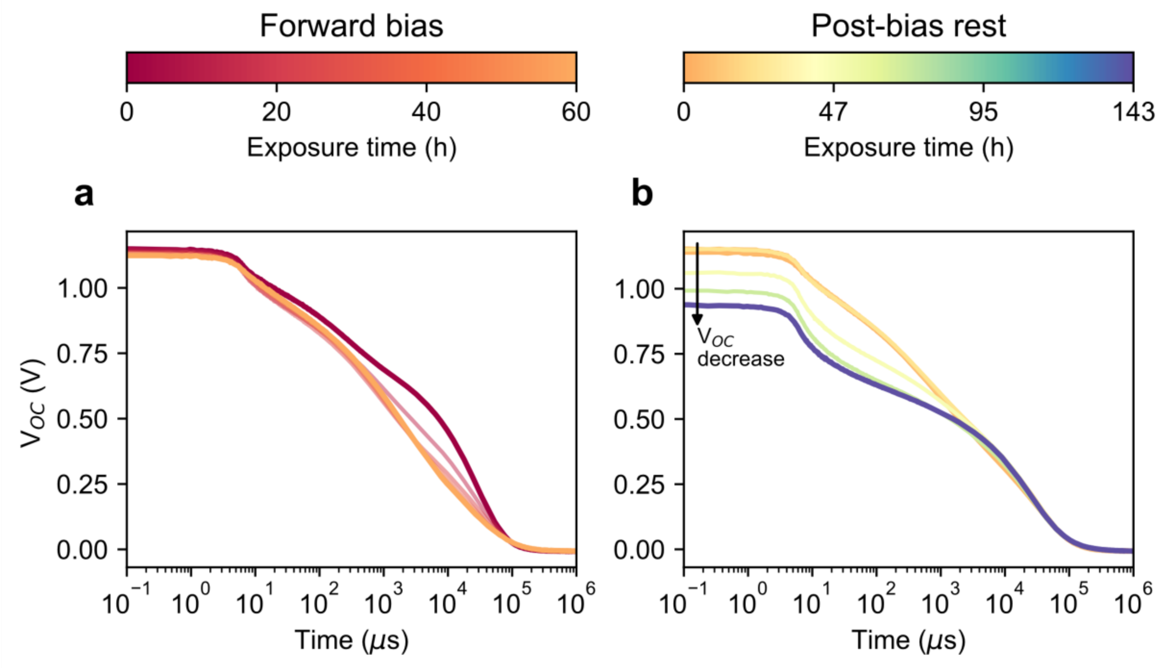

Figure S12. Evolution of non-normalized OCVD curves during forward bias **(a)** and subsequent post-bias rest **(b)** phases, highlighting the V_{OC} drop during the post-bias rest phase.

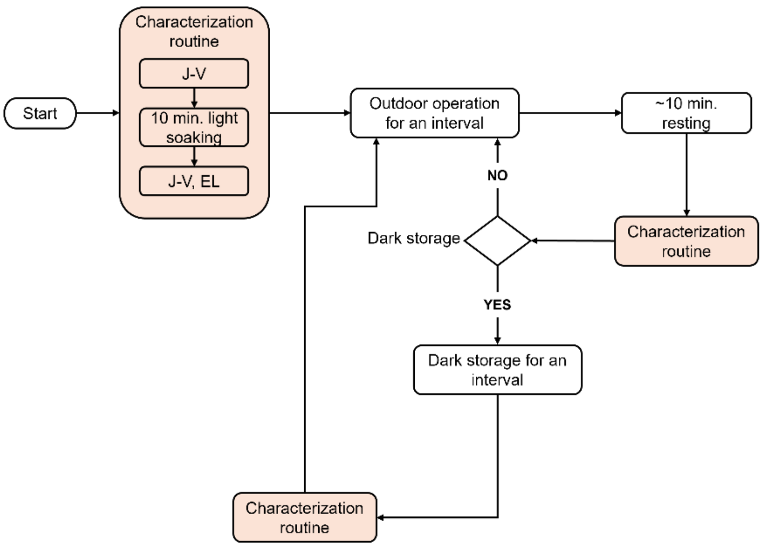

Figure S13. Workflow diagram illustrating the outdoor testing.

Supplementary Note 1: Fatigue Behavior

Cycled light (or day/night cycles outdoors) leads to a phenomenon in PSCs known as 'fatigue'[4–6], where time required to reach peak performance progressively increases with each cycle, attributed to cyclic ion migration[5]. Notably, the fatigue phenomenon is also present in cells subjected to indoor test sequence. Figure S7 shows the evolution of V_{OC} during light soaking of a representative cell at open-circuit condition under solar simulator at 1 sun intensity. Initially, the cells V_{OC} exhibited only a slight increase during the first 5 minutes of light soaking, after which it saturated. After undergoing the 1.2 V forward bias phase and subsequently resting for approximately 4000 hours in the post-bias phase, the cell was subjected to light soaking in 10-minutes intervals. Instead of reaching saturation quickly, V_{OC} showed a steady increase over the entire 30-minute light soaking period, indicating that the cell now requires a much longer light exposure to reach its peak V_{OC}.

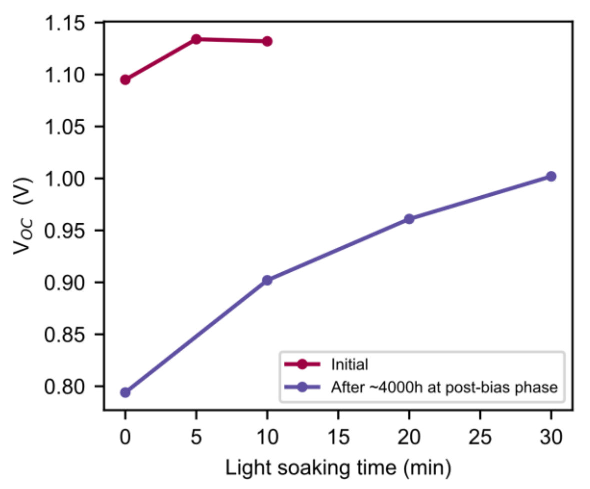

Figure S14. Fatigue behavior of a representative cell before and after the indoor test sequence.

Supplementary Note 2: Extended Outdoor Data

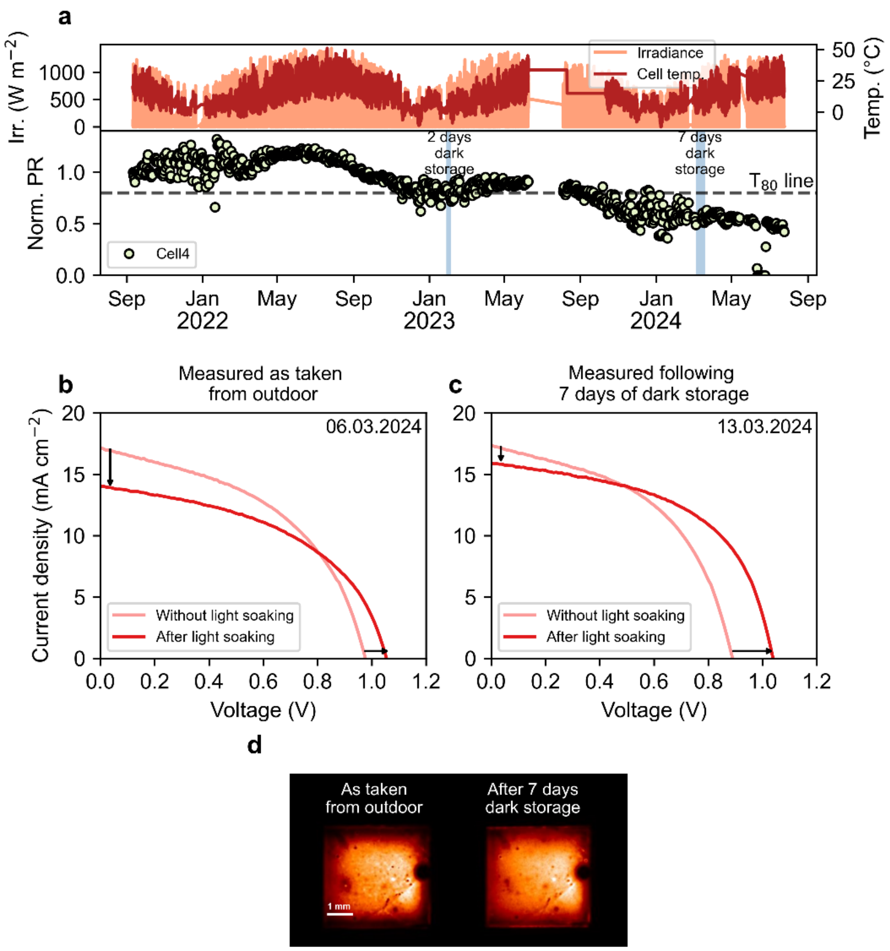

Figure S15. The adverse effect of short light soaking emerges with extended outdoor aging. **(a)** Extended irradiance and cell temperature profiles, combined with normalized PR of Cell4. **(b)** J-V curves measured after removing the cell from the outdoor setup. **(c)** J-V curves following 7 days of dark storage. **(d)** EL images of the cell before and after dark storage. Arrows indicate the changes on J_{SC} and V_{OC} upon light soaking. Note that the gap in (a) results from a data logging issue. The cell was continuously MPP-tracked for the whole period except during indoor measurements and intentional dark storage phases.

Even though the adverse effect of light soaking significantly diminished following 7 days of dark storage, we note that the drop in V_{OC} (when the states after 10 minutes of light soaking are compared Figure S15a-b) and the shrinking of the defect in EL images after dark storage (Figure S15c) did not reach the extent observed in the indoor test sequence (Figure 2) or after 2 days of dark storage to which the cell had been subjected earlier (Figure 4). This is likely due to multiple overlapping degradation-recovery mechanisms during the third year of outdoor exposure, leading to discrepancies (or delays) in the expected cell response patterns. Further supporting this hypothesis, we demonstrate another cell from a different batch (same device stack, geometry and encapsulation) in Figure S16. This cell was subjected to outdoor testing for 25 months before being removed from the outdoor setup and exposed to the same dark storage scheme as Cell4 (Figure S15). After 7 days of dark storage, this cell's V_{OC} significantly decreased, while J_{SC} and R_{SH} substantially increased (when the states after 10 minutes of light soaking are compared in Figure S16a-b). Additionally, the adverse light-soaking effect disappeared completely, and the previously grown defects in EL images changed more pronouncedly (Figure S16c), aligning much more closely with the observed features of the post-bias rest phase (Table 1). This suggests that the features associated with dynamic ion migration might be influenced by the extent of total degradation in outdoor-aged PSCs.

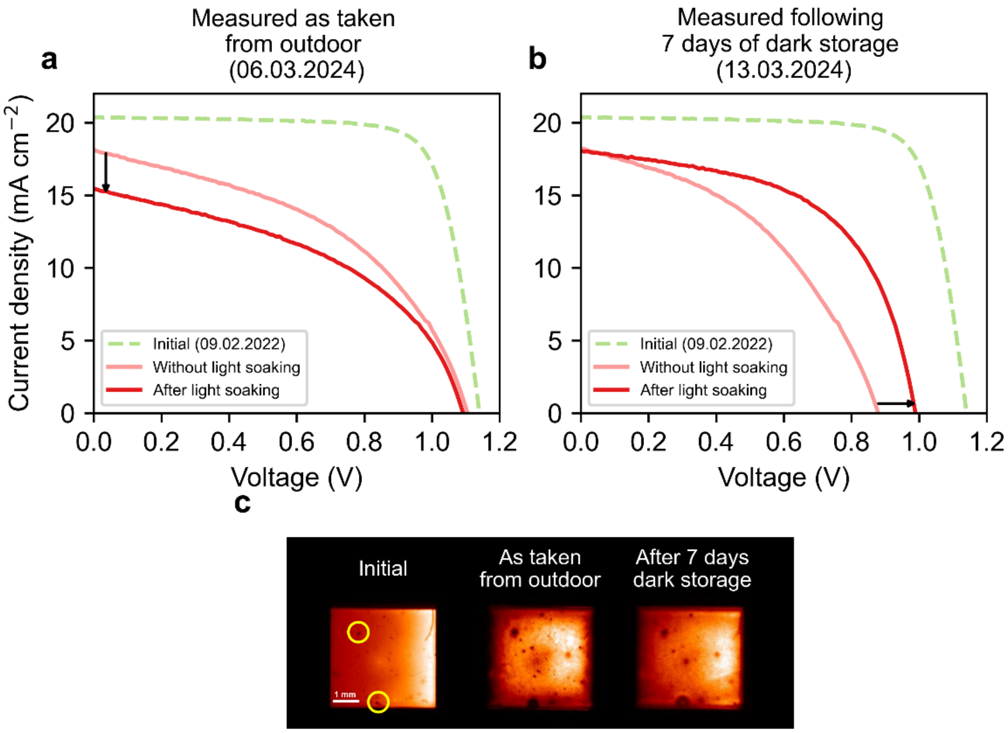

Figure S16. The 'features' on another encapsulated PSC with the same device stack, subjected to outdoor testing (ISOS-O-3, Berlin, Germany) for 25 months. This cell was installed in the outdoor setup 5 months later than the batch (Cell4) discussed in the paper. **(a)** J-V curves measured after removing the cell from the outdoor, showing a substantial J_{SC} drop after light soaking **(b)** J-V curves following 7 days of dark storage, where adverse effect of light soaking disappeared. The J-V curve before the start of outdoor test (initial) is shown in (a,b) with green dashed line as reference. Only forward scans are shown. **(c)** Evolution of EL images, revealing both the growth of preexisting defects (highlighted with the yellow circles on the initial image) after outdoor operation and changes after dark storage.

REFERENCES

(1) Erdil, U.; Khenkin, M.; Bernardes de Araujo, W. M.; Emery, Q.; Lauermann, I.; Paraskeva, V.; Norton, M.; Vediappan, S.; Kumar, D. K.; Gupta, R. K.; Visoly-Fisher, I.; Hadjipanayi, M.; Georghiou, G. E.; Schlatmann, R.; Abate, A.; Katz, E. A.; Ulbrich, C. Delamination of Perovskite Solar Cells in Thermal Cycling and Outdoor Tests. *Energy Technology n/a* (n/a), 2401280. https://doi.org/10.1002/ente.202401280.

(2) Emery, Q.; Remec, M.; Paramasivam, G.; Janke, S.; Dagar, J.; Ulbrich, C.; Schlatmann, R.; Stannowski, B.; Unger, E.; Khenkin, M. Encapsulation and Outdoor Testing of Perovskite Solar Cells: Comparing Industrially Relevant Process with a Simplified Lab Procedure. *ACS Appl. Mater. Interfaces* **2022**, *14* (4), 5159–5167. https://doi.org/10.1021/acsami.1c14720.

(3) Király, F.; Löning, M.; Bagnall, T.; Middlehurst, M.; Ganesh, S.; Ray, A.; Walter, M.; Oastler, G.; Lines, J.; ViktorKaz; Heidrich, B.; Mentel, L.; Mishra, S.; chrisholder; Bartling, D.; Tsaprounis, L.; RNKuhns; Armaghan; Gilbert, C.; Baichoo, M.; Akmal, H.; Rockenschaub, P.; Owoseni, T.; Guzal; Nshuti, F. H.; Alex-JG3; eenticott-shell; Prajapati, P.; Alavi, S. Sktime/Sktime: V0.34.0, 2024. https://doi.org/10.5281/zenodo.13955880.

(4) Huang, F.; Jiang, L.; Pascoe, A. R.; Yan, Y.; Bach, U.; Spiccia, L.; Cheng, Y.-B. Fatigue Behavior of Planar CH3NH3PbI3 Perovskite Solar Cells Revealed by Light on/off Diurnal Cycling. *Nano Energy* **2016**, *27*, 509–514. https://doi.org/10.1016/j.nanoen.2016.07.033.

(5) Jiang, L.; Lu, J.; Raga, S. R.; Sun, J.; Lin, X.; Huang, W.; Huang, F.; Bach, U.; Cheng, Y.-B. Fatigue Stability of CH3NH3PbI3 Based Perovskite Solar Cells in Day/Night Cycling. *Nano Energy* **2019**, *58*, 687–694. https://doi.org/10.1016/j.nanoen.2019.02.005.

(6) Zhang, Y.; Song, Q.; Liu, G.; Chen, Y.; Guo, Z.; Li, N.; Niu, X.; Qiu, Z.; Zhou, W.; Huang, Z.; Zhu, C.; Zai, H.; Ma, S.; Bai, Y.; Chen, Q.; Huang, W.; Zhao, Q.; Zhou, H. Improved Fatigue Behaviour of Perovskite Solar Cells with an Interfacial Starch–Polyiodide Buffer Layer. *Nat. Photon.* **2023**, *17* (12), 1066–1073. https://doi.org/10.1038/s41566-023-01287-w.
